# Reverse optogenetics of G protein signaling by zebrafish non-visual opsin Opn7b for synchronization of neuronal networks

**DOI:** 10.1038/s41467-021-24718-0

**Published:** 2021-07-23

**Authors:** Raziye Karapinar, Jan Claudius Schwitalla, Dennis Eickelbeck, Johanna Pakusch, Brix Mücher, Michelle Grömmke, Tatjana Surdin, Thomas Knöpfel, Melanie D. Mark, Ida Siveke, Stefan Herlitze

**Affiliations:** 1 Department of Zoology and Neurobiology, Ruhr-University Bochum, Bochum, Germany; 2 Laboratory of Optogenetics and Circuit Neuroscience, Imperial College London, London, UK; 3 The Francis Crick Institute, London, UK; 4 Behavioral Neuroscience, Ruhr-University Bochum, Bochum, Germany; 5 German Cancer Consortium (DKTK/DKFZ), West German Cancer Center, University Hospital Essen, Essen, Germany

**Keywords:** Epilepsy, Ion channels in the nervous system, Neural circuits

## Abstract

Opn7b is a non-visual G protein-coupled receptor expressed in zebrafish. Here we find that Opn7b expressed in HEK cells constitutively activates the G_i/o_ pathway and illumination with blue/green light inactivates G protein-coupled inwardly rectifying potassium channels. This suggests that light acts as an inverse agonist for Opn7b and can be used as an optogenetic tool to inhibit neuronal networks in the dark and interrupt constitutive inhibition in the light. Consistent with this prediction, illumination of recombinant expressed Opn7b in cortical pyramidal cells results in increased neuronal activity. In awake mice, light stimulation of Opn7b expressed in pyramidal cells of somatosensory cortex reliably induces generalized epileptiform activity within a short (<10 s) delay after onset of stimulation. Our study demonstrates a reversed mechanism for G protein-coupled receptor control and Opn7b as a tool for controlling neural circuit properties.

## Abstract (web summary)

Microbial rhodopsins can be used to control action potentials, while animal opsins can be used to control intracellular signaling pathways. The authors identify Opn7b as constitutively active G_i/o_ coupled receptor that can be deactivated by light and used to modulate neuronal activity.

## Introduction

Optogenetics provides a versatile methodology to study neuronal dynamics in a precise spatial and temporal manner in behaving animals^1^. The recombinant expression of naturally occurring and engineered light-sensitive proteins immensely contributed to our current understanding of neuronal networks, neurons and their signaling pathways. For activation and inhibition of neuronal action potential generation, microbial opsins such as channelrhodopsin-2 (ChR2), halorhodopsin or their corresponding variants have widely been used^2,3^. While microbial rhodopsins can be used to mimic fast synaptic transmission, G protein-coupled opsins can be used to simulate intracellular signaling pathways that usually act on a slower timescale. G protein-coupled receptors (GPCRs) couple to four main G protein pathways, i.e., G_i/o_, G_s_, G_q/11_, and G_12/13_. In recent years various light-activated GPCRs have been used to specifically control these signaling pathways, including vertebrate rod and cone opsins as well as parapinopsin (UVLamp) for the control of the G_i/o_ pathway^2,4,5^, vertebrate melanopsin and invertebrate rhodopsin for the control of the G_q/11_ pathway^6–8^ and jellyfish opsin for the control of the G_s_ pathway^9^.

GPCRs are commonly activated by physical stimuli or binding of a chemical ligand, where activation of the downstream G protein-mediated signal can range from milliseconds to hours. Some GPCRs exhibit an agonist-independent intrinsic constitutive activity which is poorly understood^10,11^. Alteration in the function of GPCRs coupling to the G_i/o_ pathways that typically dampen neuronal activity via activation of G protein-coupled inwardly rectifying potassium (GIRK) channels have been associated with certain forms of epilepsy^12^. To this date, no light-sensitive constitutively active GPCR has been reported and may be a valuable tool to gain new insights into the mechanisms of constitutive activity and consequences of their disruptions.

In order to precisely control diverse physiological signals, a plethora of light-activated GPCRs naturally evolved. In the zebrafish *Danio rerio* 42 opsin genes have been identified based on a functional genomic approach^13^. These genes encode 10 classical photoreceptors and 32 non-visual opsins. The phylogenetic analysis revealed 10 new opsin genes, which are divided into four new classes opn6-9. Among these new classes, opn7 is highest expressed in the brain but is also found in the eye and throughout other body tissues, including pineal, testis and muscle. Opn7 consists of four family members opn7a-d. Whole-cell recordings from Neuro2a-cells expressing Opn7b showed light-induced chromophore-dependent Ca^2+^ currents, which have been suggested to be mediated via activation of the G_q/11_ pathway^13^.

In search for constitutively active GPCRs we discovered Opn7b to be a light-sensitive constitutively active G_i/o_ coupled receptor. To characterize the biophysical properties of Opn7b, we co-expressed Opn7b and GIRK1/2 channels in HEK293 cells. After demonstrating G_i/o_ specificity and constitutive activation, we explored the functional impact of Opn7b expression in neocortical pyramidal cells (PCs). The constitutive activation of the G_i__/o_ pathway did not cause an overt behavioral phenotype, likely due to homeostatic compensatory mechanisms. However, light-induced deactivation of Opn7b led to generalized epileptiform activity (EA). Opn7b acts as a constitutively active GPCR and allows for investigating the role of these understudied GPCRs in health and disease.

## Results

### Zebrafish Opn7b constitutively activates the G_i/o_ pathway in HEK293 cells

**Fig. 1:**
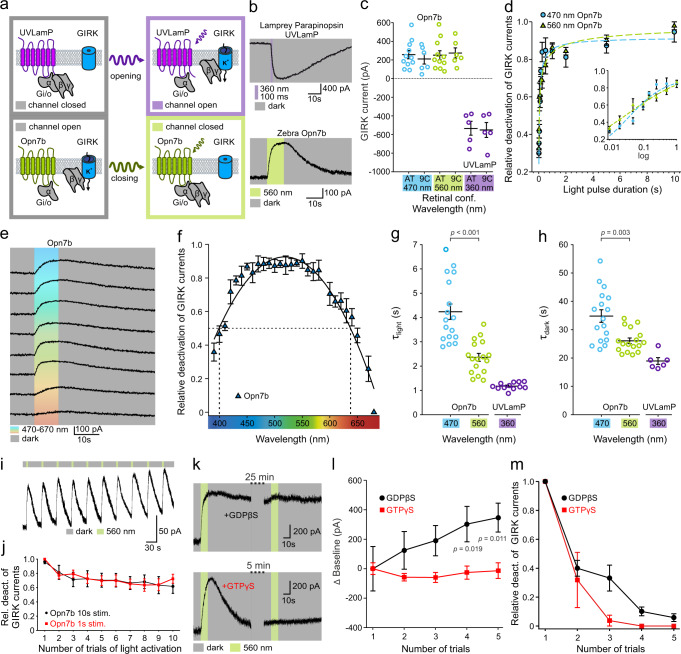
Opn7b constitutively activates the G_i/o_ pathway in HEK293 cells. **a** Schematic representation of G_i/o_ mediated (de-)activation of G protein-coupled inwardly rectifying potassium (GIRK) channels illustrating the reverted functionality of Opn7b (bottom) in contrast to classical G protein-coupled receptor (GPCR)-type opsins (top); i.e., lamprey parapinopsin (UVLamP). Typical GPCR-type opsins convert from their dark-adapted, stable resting or inactive state to their G protein-activating state upon photon absorption (monostable) or interconvert between both states in a light-dependent manner (multistable). In contrast to both groups, the monostable G_i/o_-coupled Opn7b exhibits a dark-adapted, stable G protein-activating state and is inactivated upon photon absorption. **b** Patch-clamp recording example traces of GIRK currents. The classical G_i/o_-coupled GPCR-type opsin UVLamP induces G protein-mediated opening of co-expressed GIRK channels (inward K^+^ current under given experimental conditions) upon light stimulation. In contrast, under the same experimental conditions, in darkness, G_i/o_-coupled Opn7b leads to sustained G protein-mediated opening of co-expressed GIRK channels that are closed upon light stimulation. **c** Comparison of light (de-)activated GIRK current amplitude for UVLamP (*n* = 5 cells) and Opn7b (all-trans *n* = 11 cells, 9-cis *n* = 6 cells), using all-trans (AT) or 9-cis (9 C) retinal and light of the indicated wavelengths. Mean values (±SEM) and single-cell data (circles) are shown. **d** Light pulse duration dependence of Opn7b inactivation using light of the indicated wavelengths. Mean values (± SEM) are shown (*n* = 4 cells per group). **e** Patch-clamp recording example traces of GIRK currents for Opn7b at different wavelengths. **f** Action spectrum depicting the wavelength dependence of Opn7b inactivation. Mean values (±SEM) are shown (*n* = 5 cells). **g** Comparison of light-induced GIRK current (de-)activation time constants (τlight) for Opn7b (*n* = 17 cells) and UVLamP (*n* = 12 cells) at the indicated wavelengths. Mean values (±SEM) and single-cell data (circles) are shown (two-sided Mann–Whitney-U-test). **h** Comparison of darkness-induced post-stimulus GIRK current (de-)activation time constants (τdark) for Opn7b (*n* = 17 cells) and UVLamP (*n* = 6 cells) at the indicated wavelengths. Mean values (±SEM) and single-cell data (circles) are shown (two-sided Mann–Whitney-U-test). **i** Patch-clamp recording example traces of GIRK currents for repetitive activation of Opn7b. **j** Relative GIRK current amplitude for repetitive activation of Opn7b. Mean values (±SEM) are shown (10 s stim *n* = 5 cells, 1 s stim *n* = 6 cells). **k** Patch-clamp recording example traces of GIRK currents for Opn7b under pipette-mediated (intracellular) application of guanosine diphosphate beta S (GDPβS, top) or guanosine triphosphate gamma S (GTPγS, bottom) at different time points after establishment of whole-cell configuration. **l** Comparison of GIRK current baseline for repetitive activation of Opn7b under application of GDPβS (black) or GTPγS (red) at different time points after establishment of whole-cell configuration. Mean values (±SEM) are shown (*n* = 5 cells per group; two-way repeated measure ANOVA followed by Bonferroni test, F(1,8) = 4.881, *p* = 0.058). **m** Relative GIRK current amplitude for repetitive activation of Opn7b under application of GDPβS (black) or GTPγS (red) at different time points after establishment of whole-cell configuration. Mean values (±SEM) are shown (*n* = 5 cells per group). Source data are provided as a Source Data file.

To determine the G protein specificity of Opn7b, we co-expressed Opn7b with GCaMP6 for analysis of the G_q/11_ or with Pink-Flamindo for the analysis of G_s_ or expressed Opn7b in HEK293 cells stably expressing GIRK1/2 for analysis of G_i/o_ pathway activation. In contrast to previously characterized optogenetic tools UVLamP^5^ or melanopsin^7^, which activate GIRK currents by light, we found that illumination of Opn7b deactivated GIRK currents (Fig. 1a–d). This effect is blocked by the G_i/o_ inhibitor pertussis toxin (PTX) or the GIRK channel blocker TertiapinQ (Supplementary Fig. 1), but not by the antagonist of G_q/11_ GPAnt-2A or YM-254890 (Supplementary Fig. 2). In addition, the light deactivation of Opn7b does not induce Ca^2+^ mediated GCaMP6 and cAMP-mediated Pink-Flamindo responses (Supplementary Fig. 2). These results suggest that light acts as an inverse agonist for Opn7b by constitutively activating only the G_i/o_ pathway in the dark and not interacting with the G_q/11_ and G_s_ pathways.

To analyze Opn7b/G_i/o_-dependent modulation of GIRK currents in more detail, we first determined the wavelength-dependent deactivation and found that GIRK currents are deactivated by 10 s light pulses in the wavelength range between 390–690 nm with a maximum light-sensitivity around 460–560 nm (Fig. 1f). The time course of light-dependent GIRK current deactivation was wavelength-dependent and is ⁓4 s (M = 4.09, SD = 1.16) at 470 nm and ⁓2 s (M = 2.19, SD = 0.63) at 560 nm (Fig. 1g). In contrast, the time course of GIRK current recovery in the dark is around ~33 s (M = 33.7, SD = 8.9; deactivation with 470 nm) and ~26 s (M = 26.02, SD = 4,33; deactivation with 560 nm) and is dependent on which wavelength was used to deactivate GIRK currents (Fig. 1h). These time courses are comparable to other light-modulated GPCRs such as UVLamp (Fig. 1g, h) or rhodopsin^2,4,5^. Opn7b mediated GIRK current deactivation and recovery can be repetitively elicited by 10 s light pulses with a 20% decline in response amplitude (Fig. 1i, j).

To directly demonstrate that Opn7b constitutively activates G proteins in the dark we included either GDPβS (to block the G protein transduction) or GTPγS (to activate the G protein transduction independent from Opn7b) in the patch electrode solution (Fig. 1k, m). In the presence of GDPβS baseline GIRK currents decreased while recovery and repetitive deactivation were occluded (Fig. 1k, m). In contrast, in the presence of GTPγS the steady-state (baseline) GIRK currents were non-responsive to illumination over time (Fig. 1k, m). Together these findings emphasize that Opn7b constitutively activates the G_i/o_ pathway in darkness.

### Blue light stimulation of Opn7b increases neuronal activity in vitro

**Fig. 2:**
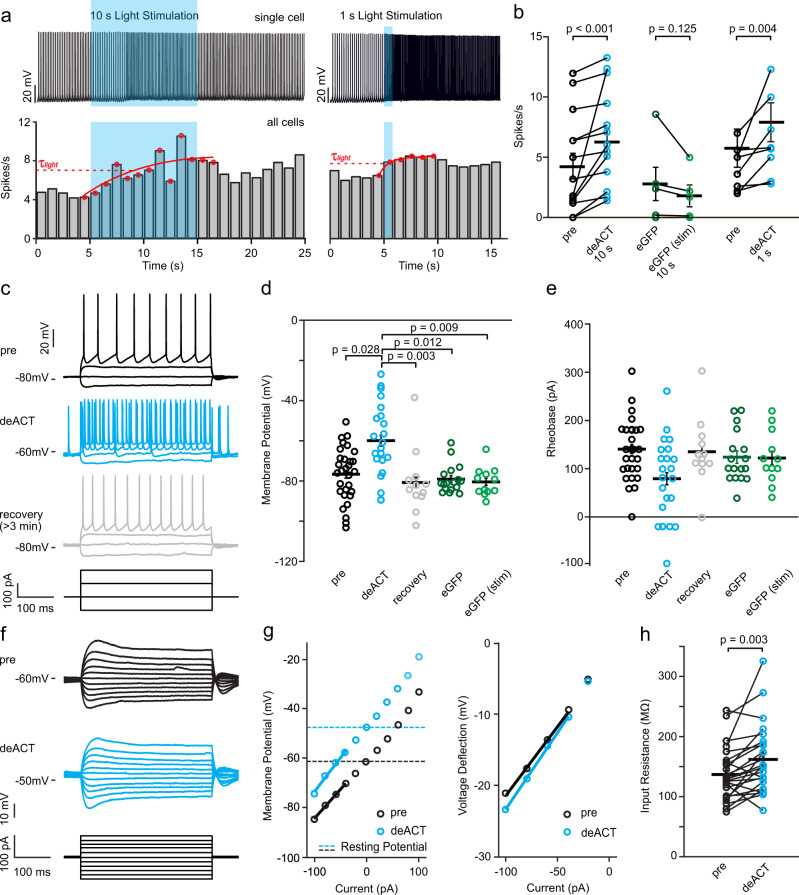
Blue light stimulation of Opn7b increases neuronal activity in vitro. **a** Example recordings of two neurons (10 s and 1 s; top). Neurons were held in current clamp (pre) and stimulated with 470 nm blue light (blue box). Light stimulation increased the firing rate of all firing Opn7b-expressing neurons (10 s: *n* = 13 cells from 11 animals and 1 s: *n* = 8 cells from 11 animals; bottom). Time constant of activation (τ_light_) of light-induced maximum firing rate is shown in red. **b** Mean response rate of Opn7b-expressing cells increased by light-induced deactivation (deACT) of Opn7b with blue light (10 s: *n* = 13 cells from 11 animals; 1 s: *n* = 8 cells from 11 animals). In contrast, light stimulation did not change the firing rate of enhanced green fluorescent protein (eGFP)-expressing neurons (*n* = 5 from 2 animals). Mean values (±SEM) and single-cell data were plotted, significance was assessed using two-sided Wilcoxon signed-rank test. **c** Example trace of a neuronal response to a −100, 0, 100, and 200 pA pulse before stimulation (pre), direct after light-induced deACT of Opn7b and after a recovery time of at least 3 min after stimulation (recovery). **d** Comparisons of membrane potentials in Opn7b-expressing neurons before (pre, black, *n* = 30 from 12 animals) and after light stimulation (blue, deACT, *n* = 22 cells from 11 animals and gray, recovery, *n* = 12 cells from 5 animals) and eGFP-expressing neurons before and after light stimulation (green, eGFP: *n* = 17 cells from 2 animals, eGFP-stim: *n* = 12 cells from 2 animals). Mean values (± SEM) and single-cell data (circles) are shown. Significance was assessed using Kruskal-Wallis-test (*p* < 0.001) followed by pairwise Dunn’s test. **e** Comparisons of rheobase in Opn7b-expressing neurons before (pre, black, *n* = 30 cells from 12 animals) and after light stimulation (blue, deACT, *n* = 22 cells from 11 animals; and gray, recovery, *n* = 12 cells from 5 animals) and eGFP-expressing neurons before and after light stimulation (green, eGFP: *n* = 17 cells from 2 animals; eGFP-stim: *n* = 12 cells from 2 animals). Mean values (±SEM) and single-cell data (circles) are shown. Significance was assessed using Kruskal-Wallis-test (*p* = 0.099). **f** Example trace of a neuronal response to various small current steps from −100 to 100 pA pulse before stimulation (pre) and direct after deACT used to calculate the input resistance. **g** I-V plot generated from the voltage response before (black) and after light-induced deactivation (blue) shown in (**f**). The average input resistance was estimated from the slope of the I-V plot between −100 and −40 pA. **h** Mean input resistance of Opn7b-expressing cells increased by light-induced deACT with blue light (*n* = 24 cells from 11 animals). Mean values (±SEM) and single-cell data were plotted, significance was assessed using two-sided Wilcoxon signed-rank test. Source data are provided as a Source Data file.

To investigate whether we could modulate the cortical circuit dynamics via the constitutively active, G_i/o_ specific Opn7b, we specifically expressed Opn7b in PCs using Nex-cre mice^14^. We performed whole-cell current clamp recordings from Opn7b expressing PCs of cortical brain slices and deactivated Opn7b using a 470 nm 1 s or 10 s light pulse (Fig. 2a, blue area). Light-mediated deactivation of Opn7b increased the rate of spontaneous action potential firing (Fig. 2a, b). The time constant to reach the maximal light-induced firing rate for all cells was between 1 s (for 1 s stimulation) to 5 s (for 10 s stimulation), while recovery from light-induced depolarization occurred within 3 min. To investigate if the increase in firing rate is correlated with a change in resting membrane potential (Vm) and the firing threshold of the PCs, we applied a train of 500 ms current pulses (Fig. 2c). We found that the Vm was around −80 mV before light stimulation (pre) and increased to approximately −60 mV upon light deactivation of Opn7b (deACT, blue) (Fig. 2d). After a recovery period of at least 3 min in the dark, the Vm returned to baseline levels of around −80 mV (recovery, gray). PCs expressing enhanced green fluorescent protein (eGFP) also revealed a Vm of about −80 mV as described previously^15–17^, but did not respond to light stimulation. We found that the rheobase, a measure of the neuronal firing threshold, was not changed in neurons after light stimulations (Fig. 2e). In addition, Opn7b expressing PCs showed an increase in the input resistance after light stimulation (Fig. 2f–h).

### Blue light stimulation of Opn7b increases single-neuron and network activity in vivo

**Fig. 3:**
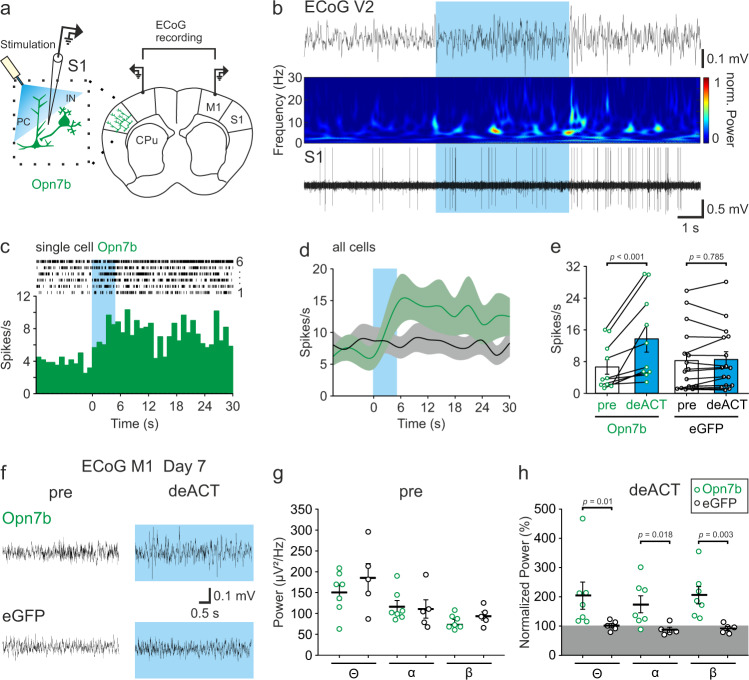
Blue light stimulation of Opn7b increases neuronal and network activity in vivo. **a** Schematic description of the unilateral light stimulation site, bilateral electrocorticographic (ECoG) recording site and simultaneous single-cell recording in awake head restrained Opn7b expressing (green) mice. Pyramidal cell (PC); Interneuron (IN); Primary motor cortex (M1); Primary somatosensory cortex (S1); Caudate putamen (CPu). **b** Example trace depicting a change in activity in the secondary visual cortex (V2) ECoG recording (top), corresponding spectral analysis (middle) and simultaneously recorded single-cell activity (bottom) in the primary somatosensory cortex (S1) after 5 s blue light (465 nm) stimulation on day 10 after injection. **c** Example raster plot (top) and peri-stimulus time histogram of a single cell in S1 (same one as depicted in B) stimulated with 5 s blue light (blue box) for 6 repetitive trials. Bin size 1 s. **d** Stimulation increased the single-cell activity in S1 neurons for Opn7b but not enhanced green fluorescent protein (eGFP; gray) expressing cells (Opn7b *n* = 10 cells from 3 animals, eGFP *n* = 16 cells from 3 animals). Solid line represents the mean, while shadowed area represents the ±SEM. **e** A significant (repeated measure two-way ANOVA followed by pairwise Bonferroni test, *F*(1,24) = 21.494, *p* < 0.001) increase in the mean spike rate (±SEM) upon blue light stimulation (deACT 12 s, blue) compared to pre-stimulation (pre 12 s, white) was found for Opn7b but not eGFP expressing cells. Each circle (Opn7b *n* = 10 cells from 3 animals, eGFP *n* = 16 cells from 3 animals) represents an individual cell, while the bar represents the mean. Error bars represented as ±SEM. **f** Example trace of ECoG activity in M1 before stimulation and during stimulation of Opn7b (top) or eGFP (bottom). **g** No changes were found in the ECoG activity in the theta, alpha and beta frequency band before stimulation in Opn7b (*n* = 7 animals) and eGFP (*n* = 5 animals). **h** A significant increase in normalized (normalized to pre activity) theta, alpha, and beta (two-sided Mann–Whitney-U-test) frequency-band activity was detectable for Opn7b (*n* = 7 animals) stimulated Bl6 mice in comparison to eGFP (*n* = 5 animals) stimulated Bl6 mice  on day 7. Each circle represents individual animals, while the bar represents the mean. Error bars represented as ±SEM. Source data are provided as a Source Data file.

Next, we investigated the effects of Opn7b expression and deactivation with light in the primary somatosensory cortex (S1) of C57/Bl6 (Bl6) mice. Opn7b was expressed in S1 PCs and interneurons using AAVs. Following 7–18 days of virus expression, simultaneous single-cell activity in the S1 and electrocorticographic (ECoG) signals in the secondary visual cortex (V2) were recorded (Fig. 3a, b). Single-cell activity in the S1 increased following unilateral 5 s blue light (465 nm) stimulation in 10 out of 14 neurons (Fig. 3c–e), lasting ~120 s (Supplementary Fig. 3). In addition to the increase in single-cell activity in S1 upon light stimulation, we found increased network activity in the adjacent M1 of Opn7b expressing animals compared to eGFP expressing control animals after bilateral light stimulation (Fig. 3f–h). This Opn7b light-induced rise in M1 network activity was detectable in all three frequency bands, theta, alpha and beta, following light stimulation (Fig. 3h). Taken together, optogenetic activation of the mouse S1 expressing Opn7b increased the single-neuron activity as well as network activity in adjacent M1 regions.

### Light-mediated deactivation of Opn7b in primary somatosensory cortex induces epileptiform activity in mice

**Fig. 4:**
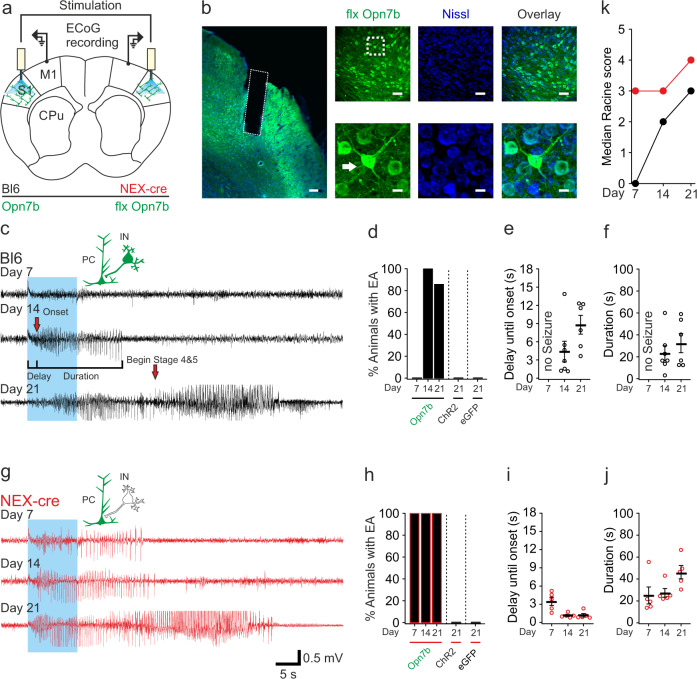
Light-mediated deactivation of Opn7b in primary somatosensory cortex induces epileptiform activity in mice. **a** Schematic description of the bilateral electrocorticographic (ECoG) recording in M1. For simultaneous light stimulation, bilateral optic fibers were implanted in the primary somatosensory cortex (S1) after viral injection. Primary motor cortex (M1); Caudate putamen  (CPu). **b** Representative example of floxed (flx) Opn7b (green) expression in S1 and general neuronal staining (Nissl, blue) in NEX-cre mice. The dashed box in the left panel represents the fiber tract of the implanted fiber. Scale bar 100 µm. The dashed box in the right top panel is a zoom of a single neuron and its axon (arrow) in the bottom panel. Scale bar 50 µm top panel and 10 µm bottom panel. **c** Bl6 mice (black trace) and (**g**) NEX-cre mice (red trace) were recorded weekly and stimulated with 10 s blue light (465 nm, blue box). **c** First epileptiform activities (EAs) were detectable on day 14 after virus injection following stimulation in Bl6 mice expressing Opn7b and (**g**) on day 7 in NEX-cre mice in ECoG recordings and video recordings. Pyramidal cell (PC); Interneuron (IN). **d** Bars represent the percentage of Bl6 mice (*n* = 7 animals) and (**h**) NEX-cre (*n* = 5 animals) mice that developed EA after light stimulation. None of the Bl6 controls expressing channelrhodopsin-2 (ChR2, *n* = 3 animals) or enhanced green fluorescent protein (eGFP, *n* = 5 animals) showed pathological ECoG changes upon light stimulation. NEX-cre mice developed EA on every recording day following floxed Opn7b stimulation, while none of the NEX-cre floxed ChR2 (*n* = 3 animals) or floxed eGFP mice (*n* = 8 animals) developed EA upon light stimulation. **e**, **i** The delay until onset of EA was measured at the time between stimulation onset and the beginning of ECoG EA (see c). Bl6 day 14 *n* = 7, day 21 *n* = 6 and Nex-cre day 7–21 *n* = 5. **f**, **j** The EA lasted around 30 s for Bl6 and NEX-cre mice. Bl6 day 14 *n* = 7, day 21 *n* = 6 and Nex-cre day 7–21 *n* = 5. **k** The severity of EA was measured as the median value of the modified Racine scale which was higher on day 7 in NEX-cre mice than in Bl6 mice. Bl6 day 7–21 *n* = 7 and Nex-cre day 7–21 *n* = 5. Each circle represents individual EA, while the bar represents the mean value for all animals. Error bars represented as ±SEM. Source data are provided as a Source Data file.

Due to the increase in network activity, we explored if stimulation of Opn7b can be a suitable tool to induce pathological network synchronicity. To test this possibility, we expressed either the constitutively active opsin Opn7b, the excitatory opsin ChR2 that modulates neuronal activity upon light stimulation via direct light-dependent cation influx, or eGFP alone as a control in S1. We monitored bilateral ECoG activity in M1 and V2 as well as mouse behavior in response to light stimulation on days 7, 14, and 21 after injection of an AAV that targets all cell types including PCs and interneurons (Fig. 4a, b). After 14 days of expression light stimulation of S1 induced primary generalized EA in all Opn7b expressing Bl6 mice (Fig. 4c, d). None of the animals expressing either ChR2 or eGFP developed pathological ECoG activity changes upon light stimulation (Fig. 4d). Opn7b induced EA was triggered by light application after a delay of 4 s on day 14 (M = 4.38, SD = 4.18) or 8 s on day 21 (M = 8.8, SD = 3.45) and lasted on average for 23 s on day 14 (M = 23.22, SD = 16.89) or 32 s on day 21 (M = 32.39, SD = 19.44) (Fig. 4e, f). Furthermore, none of the tested Opn7b expressing animals developed focal or unprovoked (non-stimulated) spontaneous EA during 24 h ECoG recordings.

Next, we tested if the epileptogenic effect of Opn7b could be attributed to a specific cell type in the S1 by expressing Opn7b only in PCs (NEX-cre). We found that light-mediated deactivation of Opn7b expressed in PCs induced primary generalized EA at day 7 in 100% of the mice (Fig. 4g, h). In contrast, the expression of ChR2 or eGFP in PCs or Opn7b in GABAergic interneurons alone did not induce any pathological ECoG abnormalities upon 10 s light stimulation (Fig. 4h, Supplementary Figs. 4 & 5). The delay until onset of the EA decreased from 3 s on day 7 (M = 3.38, SD = 1.35) to ~1 s on day 14 or 21 (day 14 M = 1.15, SD = 0.33, day 21 M = 1.18, SD = 0.57), while the duration of the EA increased from 25 s on day 7–27 s and 46 s on day 14 and 21 (day 14 M = 27.39, SD = 7.87; day 21 M = 46.23, SD = 12.13), respectively (Fig. 4i, j). Furthermore, the strength of EA induced by Opn7b deactivation, measured by the modified Racine scale, was higher in PC specific expression in NEX-cre mice compared to pan neuronal expression in Bl6 mice and escalated with increased expression time of Opn7b (Fig. 4k). The induced EA displayed classical elements of generalized epilepsy such as interictal events, postictal suppression and polyspikes in the ECoG (Supplementary Fig. 6). In the absence of light stimulation Opn7b expressing mice did not develop spontaneous EA and showed normal motor, sensory, social and memory skills (Supplementary Figs. 7 & 8). These data demonstrated that Opn7b reliably and precisely induces EA in a cell-type specific manner that show characteristics of classical seizure models.

### Calcium dynamics of Opn7b induced epileptiform activity synchronize with electrocorticogram activity

**Fig. 5:**
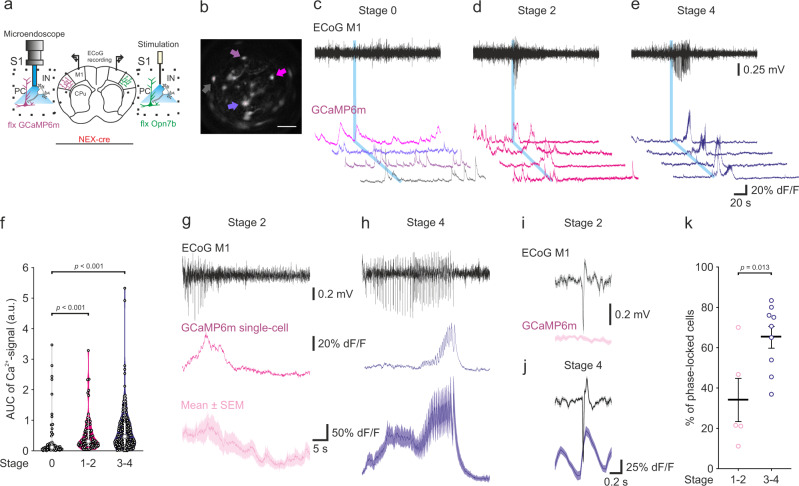
Calcium dynamics of Opn7b induced epileptiform activity synchronize with electrocorticogram activity. **a** Schematic example of unilateral injection and stimulation site for floxed (flx) Opn7b (green), bilateral electrocorticographic (ECoG) recording site and unilateral injection and recording of floxed GCaMP (purple) using a microendoscope in primary somatosensory cortex (S1) of NEX-cre mice. Primary motor cortex (M1); Caudate Putamen (CPu); Pyramidal cell (PC); Interneuron (IN). **b** Representative maximum projection of Ca^2+^ signals in S1. Colored arrows represent cells shown in C1 bottom. Scale bar 100 µm. Recording of epileptiform activity (EA, Top) and unilateral recording of Ca^2+^ activity (GCaMP6m, bottom) after blue light (465 nm, 1 s) stimulation revealed varying Ca^2+^ activity for stage 0 (**c**), 1-2 (**d**) and 3-4 (**e**) EA. **f** Area under the curve (AUC) for the Ca^2+^ signal following blue light stimulation was significantly increased for EA of stage 1–2 (*n* = 86 cells from 5 EAs in 2 animals) and stage 3–4 (*n* = 180 cells from 9 EAs in 2 animals) in comparison to stage 0 (no EA, *n* = 62 cells from 2 animals, Kruskal-Wallis-test followed by pairwise Dunn’s test, *p* < 0.001). Small black circles represent individual cells, large gray circle representing median, white box 25th and 75th percentile and whiskers 5th and 95th percentile. **g** Representative example of stage 2 EA in the ECoG recording (Top, black), the Ca^2+^ activity of an individual cell (middle) and mean activity of all recorded cells during the EA (bottom, *n* = 18 cells) and (**h**) stage 4 mean Ca^2+^ activity (*n* = 20). g and h solid line represents mean, while shadowed area represents the ±SEM. **i** Example of peak triggered average for EA (top, ECoG M1) and its corresponding average Ca^2+^ signal (bottom, GCaMP6m) showed low phase locking of the Ca^2+^ signal with the EA for stage 2 (*n* = 13 peaks, same EA as depicted in g). **j** but high phase-locking for stage 4 EA (*n* = 50 peaks, same EA as depicted in h). Solid line represents mean, while shadowed area represents the ±SEM. **k** The percentage of phase-locked cells during EA is significantly higher during stage 3–4 EA (*n* = 9 EAs from 2 animals) than during stage 1–2 EA (*n* = 5 EAs from 2 animals, *t*(12) = −2.903 two-sided independent samples *t* test). Each circle represents individual EA, while the bar represents the mean value for all. Error bars represented as ±SEM. Source data are provided as a Source Data file.

In contrast to other epilepsy models, light-mediated deactivation of Opn7b immediately triggers EA, allowing for the precise temporal control of EA onset. Thus, Opn7b induced EA is a model to study neuronal dynamics during development and propagation of EA. To further test Opn7b as a tool for epilepsy research, we investigated if Opn7b induced EA can be reliably monitored with state-of-the-art techniques such as Ca^2+^ imaging in behaving mice. First, we verified that a 1 s unilateral stimulation alone was sufficient to induce generalized EA (Supplementary Fig. 9). Simultaneous deactivation of Opn7b unilaterally and Ca^2+^ recordings (GCaMP6m) contralaterally (Fig. 5a, b) demonstrated significant increases in Ca^2+^ signals during EA compared to the absence of EA (Fig. 5c–f). We found no difference in the area under the curve (AUC) between seizure stages but seizures depicted larger AUC than no seizures (Fig. 5f). Ca^2+^ signals of individual cells often displayed synchronization with the individual ECoG peaks at stage 3–4 (Fig. 5g, h) which was also evident in the mean Ca^2+^ signal across all cells during EA (Fig. 5e bottom). To verify if individual Ca^2+^ signals were phase-locked with the ECoG peaks, we calculated a peak triggered average for each cell during EA (Fig. 5i, j). Stage 3-4 EA displayed more phase-locked Ca^2+^ activity than stage 1-2 (Fig. 5k). Unilateral Opn7b deactivation induced generalized EA and synchronized contralateral Ca^2+^ activity making Opn7b a tool to study neuronal dynamics during EA development and propagation.

### Bilateral suppression of epileptiform activity by unilateral excitation of inhibitory interneurons

**Fig. 6:**
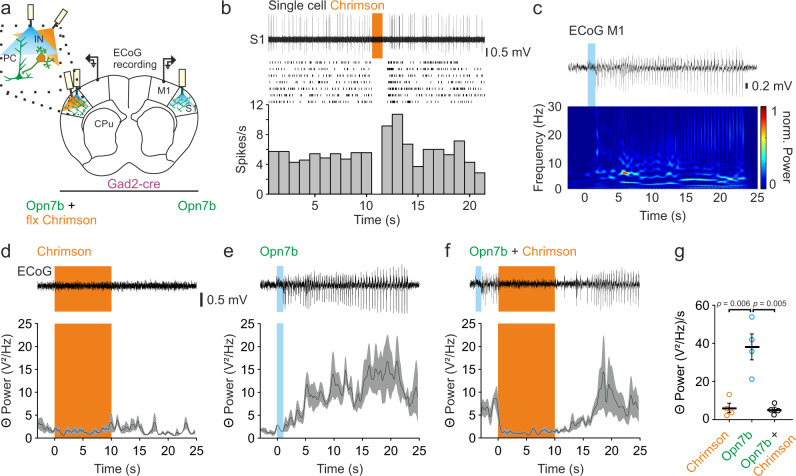
Bilateral suppression of epileptiform activity by unilateral excitation of inhibitory interneurons. **a** Schematic example of bilateral electrocorticographic (ECoG) recording, bilateral injection and stimulation site for Opn7b (green) and unilateral injection and stimulation site for floxed (flx) Chrimson (expression only in GABAergic interneurons, due to Gad2-cre animals, orange). Pyramidal cell (PC); Interneuron (IN); Primary somatosensory cortex (S1); Primary motor cortex (M1); Caudate putamen (CPu). **b** Representative example of single-cell activity of a putative PC in S1 during Chrimson stimulation of GABAergic interneurons with orange light (620 nm, 1 s; top) and corresponding raster plot (middle, 7 Trials from 1 cell) as well as peri-stimulus time histogram. Bin size 1 s. **c** Spectral characteristics of generalized epileptiform activity (EA) induced by Opn7b stimulation showed increased activity in the theta frequency band (4–8 Hz). **d** Change in mean theta power in the M1 after 10 s orange light or (**e**) 1 s blue light (465 nm) stimulation. Blue light stimulation induced generalized EA which was detectable by the increase in theta power. The top panel shows a representative ECoG trace example. **f** Mean theta power was bilaterally decreased (depicted is the contralateral side of Chrimson stimulation) during excitation of GABAergic interneurons in S1 if an EA was induced by 1 s blue light stimulation. For (**d**–**f**) bottom black line represents mean (*n* = 4) while gray area represents the ±SEM. **g** Mean theta power during 5 s of activity during orange light stimulation (no EA, *n* = 4), blue light stimulation (EA, *n* = 4) and orange light stimulation (*n* = 4) during EA (Chrimson during EA). Mean theta power during Opn7b alone was significantly higher than Chrimson alone and if Chrimson was activated after EA onset (one-way repeated measure ANOVA followed by pairwise Bonferroni test *F*(2,6) = 18.262, *p* = 0.003). Each circle represents individual animals, while the bar represents the mean value for all animals. Error bars represented as ±SEM. Source data are provided as a Source Data file.

To further evaluate the potential of Opn7b as a tool to study generalized EA, we tried to ablate the Opn7b induced EA by silencing PC activity. Therefore, we expressed and activated a second optogenetic tool (Chrimson, a red-shifted channelrhodopsin variant) in GABAergic interneurons (Gad2-cre, Fig. 6a). Activation of Chrimson in GABAergic interneurons silenced the activity of PC in the S1 during single-cell recordings (Fig. 6b). Opn7b induced EA can be identified by a strong bilateral increase in the theta (4–8 Hz) frequency band (Fig. 6c). Since the changes in theta frequency band were detectable equally on both sides, we decided to only analyze the effect in the contralateral side to the Chrimson stimulation. Unilateral activation of Chrimson and Opn7b with orange light (10 s; 620 nm) alone did not induce EA (Fig. 6d), while 1 s bilateral deactivation of Opn7b with blue light reliably induced EA indicated by a strong increase in the theta frequency band (Fig. 6e).

We next tested, if unilateral stimulation of GABAergic interneurons during EA would suppress the Opn7b induced bilateral EA. Indeed, a 10 s unilateral light-mediated stimulation of GABAergic interneurons by Chrimson after EA onset immediately suppresses the long-lasting bilateral EA during orange light stimulation (Fig. 6f). Theta frequency was comparably low during Chrimson stimulation alone but significantly increased during EA (Fig. 6g). However, if Chrimson was activated during Opn7b induced EA, theta frequency band activity decreased to normal levels. After termination of orange light stimulation EA returned. Thus, Opn7b is a constitutively active G_i/o_ coupled GPCR that may induce homeostatic inhibition of neuronal networks and reliably induces generalized EA in neocortical networks upon light stimulation, making it a tool for investigating the propagation, development and underlying neuronal network of EA.

## Discussion

We discovered that Opn7b constitutively activates the G_i/o_ pathway unless illuminated with blue/green light. Our photophysical characterization showed that Opn7b can be used as an optogenetic  tool to constitutively activate the G_i/o_ pathway and remove this activation in a transient and well-timed manner by light application. As an application example we demonstrated the potential of Opn7b as an optogenetic tool for epilepsy research. Expression of Opn7b in HEK293 cells stably expressing GIRK1/2 channels led to constitutive activation of GIRK channels demonstrating the involvement of the G_i/o_ pathway. This is in contrast with the observation that Opn7b induces a chromophore-dependent activation of an inward current in Neuro-2a cells, which has been suggested to be mediated by the G_q/11_ protein-induced stimulation of a Ca^2+^ signaling pathway^13^. Opn5L1 has recently been described as a G_i/o_ protein-coupled, reverse, self-regenerating photoreceptor that binds all-trans-retinal. Binding of all-trans-retinal to Opn5L1 stabilizes the active state of the GPCR and therefore G protein activation. Absorption of photons leads to a trans-cis isomerization of the retinal compound, causing GPCR inactivation followed by the slow self-regeneration to the active state in the dark^18^. Therefore, the activation-inactivation-reactivation cycle observed for Opn7b modulated GIRK currents resemble the reverse action of Opn5L1. According to Davies et al. (2015)^13^ the Opn7 family represents a new clade between the Opn5 and RRH (Retinal Pigment Epithelium-Derived Rhodopsin Homolog) branches. The amino acid sequence homology between the chicken Opn5L1 and zebrafish Opn7b is about 42%. Light-induced deactivation of Opn7b leads to a depolarization of somatosensory pyramidal neurons, which is correlated with an increase in the input resistance. The small change in input resistance is most likely related to the fact that deactivation of the G_i/o_ pathway leads to the modulation of various ion conductances in neurons either directly or indirectly via disinhibition of adenylyl cyclase, an increase in intracellular cAMP levels and activation of PKA. This may involve modulation of GIRK, other K^+^ channels^19,20^, TRPC^21–23^, HCN^24–26^, CNG^27^, voltage-gated Ca^2+^ channels^28^ as well as other PKA targets^29^.

Epilepsy is a severe neurological disorder characterized by repeated seizures that affect about 1% of the population^30^. The investigation of how epilepsy develops, occurs and can be treated is a major focus and challenge in epilepsy research. A key point in this challenging research are animal models, where epileptic seizures can be induced precisely and reliably. Classical models of epilepsy involve chemical (convulsant application) or electrical (kindling) induction methods applied to healthy animals. Unfortunately, these methods lack the precise control over the development and onset of seizures. Various epileptic animal models have also been developed in relation to certain proteins, brain regions and even cell types^31–33^. However, epileptic seizures still evolve spontaneously, and the starting point is difficult to control in these models. The development of optogenetic methods allow now for the cell-type-specific control of neuronal activity and therefore to switch epileptic seizures on and off^34–38^ (for overview see^30^). For Opn7b a  one second blue light pulse is sufficient to induce only a single seizure with a latency of 1–5 s lasting for 10–70 s. No reoccurring seizures or unprovoked seizures were observed. The seizure onset and duration correlates well with the time constants of Opn7b induced deactivation (~4 s) and recovery (~35 s) of GIRK currents in HEK293 cell and the Opn7b induced increase (~1–5 s) and recovery (~90 s) in firing rate in brain slices and in vivo. However, we do not know at this point, which ion channels targets are involved (see above).

So far in all seizure models, seizure induction is time-consuming and demands long-lasting light activation protocols with unreliable onset of seizures. For example, expression of ChR2 in mouse primary motor cortex required 10–15 optical stimuli to induce the first seizure, and subsequent seizure induction is only around 70%. In addition, the onset of electrographic and behavioral seizure activity was delayed by 1–2 min following optical stimuli^36^. In contrast expression of Opn7b in S1 PCs but also unspecific in the S1 reliably (100%) induces generalized seizures with a delay of onset of <3 s using a 10 s light pulse. The tool most likely also allows to investigate the factors that contribute to the development of epilepsy (epileptogensis) without inducing seizures that alter the system. As mentioned above ChR2 or kindling models of epilepsy need repetitive stimulation to alter the system’s state and success of the epilepsy model can only be identified by the seizure itself. However, these seizures can affect the system in a way that alteration in neuronal networks are induced by the seizure itself and are not related to the epileptogenesis. Thus, Opn7b is a promising optogenetic tool to control and investigate epileptic seizures. Furthermore, since Opn7b constitutively stimulates the G_i/o_ pathway in the dark, and constitutive activation can be switched off by light, Opn7b should allow for the characterization of the biological consequence of constitutive activity in health and disease.

The constitutive activity of Opn7b is expected to alter the homeostatic state of the cell over a long period of time leading to a new stable steady-state of the intracellular signaling network. Compensation of the constitutive G_i/o_ activation most likely involves the up- and down regulation of the expression of other proteins including ion channels (see for example^39^). Since various behavioral paradigms are unaffected (Figs. S7 and S8), the compensatory changes upon constitutive activation of the G_i/o_ signaling pathway in neuronal networks by Opn7b seem adequately adapted for normal network function. However, fast reversal of the adapted state by light-induced deactivation of the G_i/o_ pathway releases the cell from its new, stable homeostatic state. This leads to a more excitable state and finally to epileptiform activity. Thus expression of Opn7b in neuronal networks gives us the opportunity to analyze the homeostatic adaptation of the network by using state-of-the-art proteomic and sequencing techniques to understand the evolution and failures of homeostatic processes during epileptogenesis^40^. While Opn7b is most likely not suitable for investigating the native state of the cell, it can be used for understanding the mechanisms underlying constitutively active GPCR pathways in disease states such as 5-HT receptors in psychiatric disorders^41^ or rhodopsin in congenital stationary night blindness^42^. Constitutive GPCR activity also plays an important role in viral-mediated diseases such as cancer. Viruses such as the Kaposi’s sarcoma-associated virus or the human herpesvirus 4 and 5 use constitutive active GPCRs to hijack the cellular signaling cascades^43,44^. Furthermore, during aging and neurodegeneration levels of G_i/o_ coupled GPCRs are drastically reduced in various brain regions including frontal cortex and hippocampus^45^. Opn7b may therefore also be used to test if cell-type and brain region-specific constitutive activation of G_i/o_ pathways modulates aging and neurodegeneration and if disturbing the new adapted state by light is associated with disease phenotypes in animal models. Thus, Opn7b is a research tool to investigate constitutively active GPCR signaling pathways during altered and disease states in a temporal and cell-type specific controlled manner.

In summary, light acts as an inverse agonist for Opn7b, which constitutively activates G_i/o_ proteins in the dark and deactivates them upon light stimulation. The constitutive activation of G_i/o_ proteins leads to a homeostatic inhibition of neuronal networks, where light stimulation leads to a withdrawal from inhibition inducing generalized EA. More importantly, Opn7b can be used as a cell-type-specific tool to precisely induce EA by a short light pulse and has the potential to study diseases related to constitutive activity of GPCRs such as psychiatric diseases and cancer.

## Methods

### Generation of plasmid constructs

To construct adeno-associated virus (AAV) expression vectors and allow for the necessary large packaging capacity, the pAAV-CW3SL-eGFP vector (GenBank accession number: KJ411916.2) was used as the backbone plasmid for all Opn7b constructs^46^. The zebrafish Opn7b (*Danio rerio* novopsin-4, GenBank accession number: KT008418.1, as submitted to GenBank in 2015^13^) cDNA was inserted into the vector removing the stop-codon and adding a C-terminal eGFP as a fluorescence marker. All constructs containing this cDNA are named zebrafish Opn7b or Opn7b throughout the manuscript. Each element was PCR amplified with 16 bp overhangs and inserted into the backbone via AQUA Cloning^47^ (see Supplementary Table 1). Mouse melanopsin and lamprey parapinopsin (UVLamP) were used unmodified, as described in our previous publications^5,7^. The green and red Ca^2+^ sensors GCaMP6m and jRCaMP1b as well as the red cAMP indicator Pink Flamindo were used unmodified as described in their respective publications^48–50^. Channelrhodopsin-2 H134R (ChR2-H134R, or short ChR2) and the red-shifted channelrhodopsin Chrimson were also used unmodified as described in their respective publications^51,52^. As described in the corresponding figures, constructs without fluorescence tag, appropriate promoters for cell-type-specific expression and floxed variants for Cre-dependent expression were generated via restriction enzyme digest and ligation or PCR amplification of the initial construct, depending on experimental needs.

### Cell culture and in vitro imaging

Human embryonic kidney (HEK) tsA201 cells (Sigma Aldrich, Taufkirchen, Germany) and HEK GIRK 1/2 cells (HEK293 cells stably expressing GIRK1/2 subunits, kindly provided by Dr. A. Tinker UCL London, GB) were maintained at 37 °C in Dulbecco’s modified Eagle’s medium, 4.5 g/l D-glucose, supplemented with 10% fetal bovine serum (Gibco) and penicillin/streptomycin in a humidified incubator under 5% CO_2_. Growth medium of stable cell lines was supplemented with G418 (5 mg/ml). Cells were cultured on 35 mm glass-bottom dishes (for imaging) or plastic bottom dishes (for electrophysiology). As described in the corresponding figures, cells were single- or co-transfected with the different constructs via FuGENE® HD (Promega) according to the manufacturer’s protocol and incubated for 18–24 h before recordings. For opsin experiments 9-cis or all-trans-retinal was added to a final medium concentration of 1 µM. To image Ca^2+^ signals in HEK tsA201cells via GCaMP6m or jRCaMP1b, cells were transiently co-transfected with jRCaMP1b or GCaMP6m. Cells were seeded into poly L-lysine coated 35 mm glass-bottom dishes, transfected at 70% confluency with equal amounts of plasmid DNA and used the next day. Ca^2+^ and cAMP imaging were performed at an inverted Leica TCS SP5 confocal laser-scanning microscope, (Leica DMI6000 B, Wetzlar, Germany) interfaced to a personal computer, running Leica Application Suite Advanced Fluorescence software (LAS AF 2.6). A 20X/0.7NA objective was used to acquire time-lapse images (512 × 512 pixels with 1.2 s interval for live cell imaging). Cells were visualized via mCh or eGFP fluorescence with the 561 nm or 476 nm laser lines. Mouse melanopsin was activated and GCaMP6m was monitored with the 476 and 495 nm argon laser lines, whereas Opn7b was activated/deactivated and GCaMP6m was monitored with the 476 and 495 nm argon laser lines or Opn7b was activated/deactivated and jRCaMP1b or Pink Flamindo was monitored with the 405 nm, 476 and 561 nm laser lines. The exact stimulation protocols, including indication of dark phases without light stimulation, are shown in the corresponding figures. The adenylyl cyclase activator forskolin (Tocris, 100 µM) was bath applied at the last step of each stimulation. To exclude G_q_ protein-coupled pathway involvement, further recordings using 40 µM GPAnt-2a (internal solution) or 100 nM YM-254890 (external solution) were conducted. Fluorescence intensity of the respective sensor signal was measured over time for individual cells, normalized and scaled to the maximal response amplitude. Captured images were transferred into ImageJ software (v1.52o; NIH) and analyzed with the time series analyzer V3 plugin.

### In vitro electrophysiology

For GIRK channel recordings constructs were expressed in HEK GIRK 1/2 cells (see above). Cells were cultured on 35 mm dishes and recorded in dark room conditions after transfection. GIRK-mediated K^+^-currents were measured and analyzed as described in the following (see also^2^). The external solution was as follows: 20 mM NaCl, 120 mM KCl, 2 mM CaCl_2_, 1 mM MgCl_2_, 10 mM HEPES-KOH, pH 7.3 (KOH). Patch pipettes (2–5 MΩ) were filled with internal solution: 100 mM potassium aspartate, 40 mM KCl, 5 mM MgATP, 10 mM HEPES-KOH, 5 mM NaCl, 2 mM EGTA, 2 mM MgCl_2_, 0.01 mM GTP, pH 7.3 (KOH). Cells were recorded in external solution containing 1 µM 9-cis or all-trans-retinal (Sigma Aldrich, Taufkirchen, Germany). Experiments were conducted with an inverted microscope (Axiovert, ZEISS) and patch pipettes were controlled with a multi-micromanipulator (MPC-325, SUTTER INSTRUMENT). Transfected cells were visualized and Opn7b was manipulated with a monochromator system (Polychrome V, TILL Photonics). The exact stimulation protocols, including exact wavelengths of light stimulation and indication of dark phases without light stimulation, are shown in the corresponding figures. For the characterization of Opn7b wavelength dependence and light pulse duration dependence protocols were pseudorandomized. The exemplary lamprey parapinopsin (UVLamp) trace has been adapted from our previous publication^5^. To verify GIRK channel or G_i/o_ mediated pathway activation, measurements were repeated firstly with 200 ng/ml PTX, a G_i/o_ mediated pathway inhibitor, and then after with 1 nM TertiapinQ, a specific GIRK channel blocker, respectively. To exclude G_q_ protein-coupled pathway involvement, further recordings using 40 µM GPAnt-2a (internal solution) or 100 nM YM-254890 (external solution) were conducted.

Whole-cell patch clamp recordings of HEK cells were performed, digitized at 10 kHz and filtered with an EPC10 USB amplifier (HEKA). Series resistances were partially compensated between 70 and 90%. The PatchMaster software (v2x52, HEKA) was used for monochromator and voltage controls as well as data acquisition, and offline analysis was made with Igor Pro software (v6.36, Wavemetrics).

### AAV virus production

Recombinant AAV stocks of serotype 9 were produced via the triple-transfection method^53^ and purified using chloroform (described in^54^). In short, HEK293T cells were transfected with the vector of interest, the serotype plasmid and helper plasmid using polyethylenimine. Seventy-two hours after transfection, cells were harvested via low-speed centrifugation. Cells were resuspended in lysis buffer (150 mM NaCl, Tris-Cl pH 8.5), freeze-thawed 5–7 times and incubated with DNaseI plus MgCl_2_ at 37 °C for 30 min. PEG-8000 (10% final w/v) was added to the supernatant and the mixture was incubated for 2 h at 4 °C. After centrifugation at 3700 × *g* for 20 min at 4 °C, the PEG-precipitated pellet was resuspended in the clarified cell lysate. For purification, the resuspension was incubated with PEG-8000 for ≥1 h at 4 °C, centrifuged (3700 × *g*, 4 °C, 20 min), and the pellet resuspended in 50 mM HEPES buffer. Afterward, room-temperature chloroform (1:1 volume) was added, the mixture vortexed and then spun down at 370 × *g* at RT for 5 min. The aqueous phase was collected, filtered using a syringe filter (0.22 µm) and concentrated using PEG-8000. The concentrated AAV was resuspended in 1X PBS with 0.001% Pluronic F68, aliquoted and stored at −80 °C.

### Animals

All experiments were conducted with approval of a local ethics committee (Bezirksamt Arnsberg) and the animal care committee of Nordrhein-Westfalen (LANUV; Landesamt für Umweltschutz, Naturschutz und Verbraucherschutz Nordrhein-Westfalen, Germany; AZ. 84-02.04.2014.A203 und AZ. 81-02.04.2019.A228). The study was carried out in accordance with the European Communities Council Directive of 2010 (2010/63/ EU) for care of laboratory animals and supervised by the animal welfare commission of the Ruhr-University Bochum. Experiments were performed in 2–6 month old male and female mice of the following lines: C57Bl6/J (JAX stock no. 000664), heterozygous NEX-Cre^14^, heterozygous Gad2-Cre (Gad65-Ires-Cre mice (Gad2tm2(cre)Zjh/J), JAX stock no. 010802^55^). Mice were kept with a 12/12 day/night cycle and were housed with water and food ad libitum in individually ventilated cages (at 22 °C and 50% humidity). The genetic background of each mouse was tested by PCR with genomic DNA from tail biopsy (see Supplementary Table 1).

### Acute slice recordings

Acute coronal 350 µm thick brain slices of somatosensory cortex were cut from adult NEX-Cre mice 7 days after AAV9 injection and recordings were performed as follows. Briefly, mice were anesthetized with isoflurane and decapitated. The somatosensory cortex was sliced using a vibratome (VT1000S, Leica) in ice-cold artificial cerebrospinal fluid containing 87 mM NaCl, 2.5 mM KCl, 75 mM Sucrose, 1.25 mM NaH_2_PO_4_, 25 mM NaHCO_3_, 0.5 mM CaCl_2_, 3.5 MgCl_2_, 20 mM D(+)-Glucose with continuous oxygenation 95% O_2_ and 5% CO2. Slices were then stored for 30 min at 34 °C in the external solution containing 120 mM NaCl, 2.5 mM KCl, 2.5 mM CaCl_2_, 1.3 mM MgSO_4_, 1 mM NaH_2_PO_4_, 26.2 mM NaHCO_3_, and 20 mM D(+)-Glucose. Fluorescent eGFP-positive cells were visually identified under an upright microscope (DMLFSA, Leica) equipped with a monochromator system (Polychrome IV, TILL Photonics) flashing excitation light (light intensity, 1.6 mW/mm^2^ for 470 nm). Slices were preincubated for at least 30 min and continuously perfused with the external solution including 25 µM 9-cis-retinal, 0.025% (±)-α-tocopherol (Sigma Aldrich, Taufkirchen, Germany), 0.2% essentially fatty acid-free albumin from bovine serum (Sigma Aldrich, Taufkirchen, Germany). Whole-cell recordings were obtained from pyramidal cells in layers IV-VI. To avoid effects of the eGFP excitation, recording started after at least 15 min of recovery. All recordings were performed at 37 °C in the dark except for using infrared light filter (>700 nm) to target the cell. Patch pipettes (2.5–5 MΩ) were filled with internal solution with the composition 125 mM potassium gluconate, 4 mM NaCl, 2 mM MgCl_2_, 10 mM HEPES, 0.2 mM EGTA, 4 mM Mg-ATP, 0.4 mM Na-GTP, and 10 mM Tris-phosphocreatine, pH 7.3 (KOH). Membrane currents and voltages were recorded with an EPC10/2 amplifier (HEKA). The signals were filtered at 3 kHz and digitized at 10 kHz. Pyramidal cells were illuminated with 470 nm to stimulate Opn7b. One trial includes 5 s baseline recording in darkness, 5 to 10 s light stimulation, followed by 10 s recordings in darkness. To determine intrinsic properties, currents steps (−100 to 300 pA) with an increment of 20 pA were injected. The PatchMaster software (v2x52,HEKA) was used for the control of voltage and data acquisition, and off-line analysis was made using Matlab.

### Histology and Nissl staining

After the last recording session mice were sacrificed for histological analysis of correct viral expression. Briefly, mice were anesthetized and transcardially perfused with PBS and 4% paraformaldehyde (PFA; Sigma Aldrich, Taufkirchen, Germany) in PBS (pH 7.4). Dissected brains were post-fixed for 1–3 hrs in 4% PFA and cryoprotected in 30% sucrose (wt/v; Sigma Aldrich, Taufkirchen, Germany) in PBS at 4 °C for 2 days. Forty micrometer thick coronal sections were collected in PBS and permeabilized for 30 min in 0.3% PBS-T (0.3% Triton-X (v/v) in PBS). Selected sections were washed twice in PBS for 5 min and stained against Nissl by incubation in PBS with 640/660 or 435/455 Neurotrace (1:300; Thermo Fisher Scientific, Waltham, USA) for 20 min. After washing in PBS for 5 min brain sections were embedded in Roti®mount Fluocare (CarlRoth, Karlsruhe, Germany). Images were captured using a Leica TCS SP5II inverted confocal laser scanning microscope (Leica DMI6000 B, Wetzlar, Germany) with a 20x/0.7 NA objective. For excitation 405 nm (Neurotrace 435), 488 nm (eGFP), 561 nm (mCherry) and 630 nm (Neurotrace 640) were used. Images were later analyzed using ImageJ software (v1.52o; NIH).

### Virus injection and optical fiber implantation

Mice were anesthetized with isoflurane (initial dose: 5% in 1.1 L/min air and 1.3–2.0% in 1.1 L/min air for maintenance), placed in a stereotaxic frame (Narishige, Tokyo, Japan) and received an injection of carprofen (2 m/kg) and buprenorphine (0.1 mg/kg) for analgesia. During surgery the body temperature was controlled with a heating pad and to avoid corneal drying the eyes were coated with eye ointment. The skin was opened along the midline and the skull was exposed, cleaned and treated with Optibond All-in-One (Kerr GmbH, Rastatt, Germany). One or two small craniotomies (AP (relative to lambda): +1.25 mm ML: ± 3 mm) were made and a custom-made glass pipette attached to a 5 ml syringe was lowered to −1.2 mm. 200–250 nl virus (Table 1) was injected per side in 100 µm intervals (last injection at 0.6 mm) using pressure injection. For optogenetic stimulation custom-made optical fibers (ferrule 1.25 mm outer diameter, 230 µm bore size (CFLC230-10; Thorlabs, Dortmund, Germany); fiber diameter 200 µm, 0.39 NA, FT200EMT; Thorlabs, Dortmund, Germany) were bilaterally inserted into the craniotomy and secured perpendicular at −0.8 mm with a light-curing hybrid composite (Charisma; Heraeus Kulzer, Hanau, Germany). Animals that were used for in vivo extracellular single-cell recordings or Chrimson stimulation received an optical fiber that was inserted with an angle of 30–40° through an additional craniotomy (AP (relative to bregma): −1.8 mm ML: +3 mm). Virus injections on the site of the Chrimson stimulation contained an equal quantity of virus containing unfloxed Opn7b and floxed Chrimson (see Table 1).

**Table 1:** List of adeno-associated virus for in vivo expression.

Virus
AAV9-CMV-Opn7b.eGFP-CW3SL
AAV9-DIO-CMV-Opn7b.eGFP-CW3SL
AAV9-CMV-ChR2.mCh-CW3SL
AAV9-DIO- ef1α-ChR2.mCh
AAV9-DIO-CMV-Chrimson.tdtomato-CW3SL
AAV9-CMV-eGFP-CW3SL
AAV9-DIO-CMV-eGFP-CW3SL
AAV9-DIO-ef1α-GCaMP6m-CW3SL

### Electrode implantation and recording chamber construction

Five additional small craniotomies were used to insert ball tip gold wire electrodes (diameter: 0.15 mm; C. Hafner; Pforzheim, Germany) soldered to a microminiature connector (Digi-Key, Munich, Germany). Four electrodes were bilaterally implanted in the subdural space over the primary motor cortex (+1.5 mm AP, ±1.5 mm ML relative to bregma) and secondary visual cortex (+2.0 mm AP, ±2.2 mm ML relative to bregma). A grounding electrode was positioned on the lateral bone and an electrode that served as a reference was placed in the occipital bone (−2 mm AP, 0 mm ML relative to lambda). Muscle activity was monitored during recordings by placing an electrode in the neck muscle. For extracellular single-cell recordings one craniotomy (diameter 3 mm) without disrupting the dura mater was made at AP (relative to bregma): +1.25 mm ML: +3 mm using a microdrill. A recording chamber was constructed on the edge of the craniotomy with a light-curing hybrid composite (Charisma; Heraeus Kulzer, Hanau, Germany) and the tissue was covered with Kwik-Cast™ (World precision instruments, Sarasota, USA). For head fixation during extracellular single-cell recordings a pedestal was secured on the head. In all mice the incision was closed by embedding the electrodes with a light-curing hybrid composite (Charisma; Heraeus Kulzer, Hanau, Germany) and dental acrylic. Mice were allowed to recover at least 7 days before training or recording. Mice that underwent head fixation received prior to the first recording 3 consecutive days of training on the setup.

### In vivo electrophysiological recordings

The electrocorticogram and extracellular single-cell activity were recorded using Spike 2 (v7) software (Cambridge Electronic Design Ltd; Cambridge, UK). ECoG signals were amplified by 5000 and bandpass filtered with 3–100 Hz (DPA-2FX, npi electronic GmbH; Tamm, Germany) and sampled at 400 Hz (CED 1401, Cambridge Electronic Design Ltd; Cambridge, UK). Extracellular single-cell recordings were bandpass filtered 0.3–3 kHz, amplified 2000–4000 (EXT-02F/1, npi electronic GmbH; Tamm, Germany) and sampled at 20 kHz (CED 1401, Cambridge Electronic Design Ltd; Cambridge, UK). During extracellular single-cell recordings mice were awake and head-fixed but able to move all limbs freely on a treadmill. ECoG activity was recorded bilaterally in the V2 during extracellular recordings. Before recording the Kwik-Cast™ was carefully removed and the craniotomy was cleaned. Custom made borosilicate glass capillaries (OD 1.5 mm, ID 0.86, resistance 1–3 MΩ (BF150-86-10, Science products, Hofheim, Germany)) filled with 2 M NaCl were used to advance through the open craniotomy using a micromanipulator (MPC 200, Sutter Instruments, Novato, USA). Once a stable cell recording was identified a 5 s blue light (465 nm LED 1.5–2 mW at the tip of the fiber; Plexon, Dallas, USA) pulse was delivered through the angled optical fiber. Recordings lasted no longer than 4 h per day and were performed between days 7 and 18. For Chrimson stimulation during extracellular recordings a 1 s orange light (620 nm) pulse was delivered through the angled optical fiber.

### In vivo induction and suppression of epileptiform activity

During induction of EA video and ECoG activity were recorded for offline analysis. After connection to the headstage mice were placed in a 30 × 30 × 40 cm recording arena and allowed to freely explore the arena during the recording. After baseline recording a 10 s blue light (465 nm LED 1.5–2 mW at the tip of the fiber; Plexon, Dallas, USA) pulse was used either bilaterally or unilaterally to induce EA. Offline analysis was performed using a custom-written Matlab script and EA was identified manually based on the characteristic ECoG and video patterns. Delay until onset was the time from the beginning of the stimulation until the first epileptiform potentials were visible in the ECoG recording (Onset). Duration of each EA was the time from onset until no epileptic potentials were visible in the ECoG. EA was scored based on ECoG activity and video recordings with the help of a modified Racine scale (Table 2). For Chrimson stimulation an additional orange LED (620 nm; 1.0–2 mW at the tip of the fiber; Plexon, Dallas, USA) was connected to the angled optical fiber. Recordings were included if 1 s of blue stimulation was sufficient to induce EA. Orange light (10 s) was manually activated once EA was detectable in the ECoG recording. For induction of optogenetically induced seizures using Channelrhodopsin-2 the protocol was based on the described protocol by Khoshkhoo et al^36^. Briefly, blue light (465 nm LED 1.5–2 mW at the tip of the fiber; Plexon, Dallas, USA) stimulation with 40 Hz for 10 s was delivered approximately every 2 min and 50 s (total duration per stimulation interval ~3 min). The 10 s stimulation consisted of 400 intervals each lasting 0.025 s (0.013 on 0.012 s off). The stimulation interval was repeated 20 times or until EA developed.

**Table 2:** Modified racine scale.

Score	Behavior	EEG
1	Sudden behavioral arrest, motionless staring with orofacial automatism (mouth and facial movements)	Single High amplitude activity/slow waves
2	Head nodding with severe facial clonus	Spikes, sharp waves
3	Unilateral or bilateral forelimb clonus without rearing	Spikes or poly spikes, sharp waves
4	Bilateral forelimb clonus with rearing	Spike bursts/spike and wave discharges
5	Wild running, jumping, vocalization and severe clonus	Spike bursts/spike and wave discharges

### In vivo calcium imaging

For Ca^2+^ signal recordings virus injections were performed as described with unilateral injection of floxed Opn7b and contralateral injection of floxed GCaMP6m. A single optical fiber was inserted perpendicular in the injection site for floxed Opn7b at −0.8 mm. Microendoscopes (4.2 mm lens, Inscopix, Palo Alto, CA, USA) were placed in the injection site for floxed GCaMP6m at −0.8 mm to −1.00 mm. All data were processed and analyzed using the data processing software of Inscopix in combination with custom-written Matlab scripts. Induction of EA in mice was performed as described. Individual EA was induced by 1 s or 10 s blue light (465 nm 1.5–2 mW at the tip of the fiber) stimulation during recordings. A window of 50 s from the onset of stimulation was used to calculate the area under the curve for each cell. For analysis of phase-locking ECoG peaks were detected threshold-based for each EA. A peak triggered average (PTA) was calculated using the mean activity of all ECoG peaks during the EA (±500 ms ECoG activity surrounding the maximum of each peak). Similarly, corresponding Ca^2+^ averages were calculated for each cell that was recorded during the EA and normalized by subtraction of mean Ca^2+^ activity for each cell during the PTA. To form a baseline activity for each EA 1000 random PTAs were generated for each cell. Random data points were selected between the first and the last ECoG peak and used to generate a random PTA with an equal number of repetitions (number of Peaks) as used for the corresponding EA (shuffled data). Based on these shuffled average ECoG peaks a Ca^2+^ average was calculated as described. In order to detect phase-locking in individual cells the amplitude between the highest and lowest data point in the Ca^2+^ average surrounding the ECoG peak (±200 ms) was calculated for the Ca^2+^ activity (peak ratio) and the corresponding shuffled data (peak ratio-shuffled). Then a z-score was calculated using (peak ratio − μ_peak ratio-shuffled_)/σ_peak ratio-shuffled_. μ_peak ratio-shuffled_ is defined as the mean peak ratio for the shuffled data and σ_peakratio shuffled_ defined as the standard deviation for the shuffled data. A cell was considered phase-locked if the z-score was higher than 1.96.

### Analysis of electrophysiological data

Single-cell recordings for Opn7b were included if the activity was well-isolated, stable (at least 30 s before stimulation) and considered increased after light stimulation if the mean activity after stimulation (deACT, 12 s starting from the beginning of the 5 s stimulation) was repetitively (≥2) increased in comparison to before (12 s) stimulation. The mean activity for each stimulus was calculated from the peristimulus time histogram (bin size 3 s). Only the first stimulation was included in the analysis for changes in firing frequency. Individual action potentials were detected using a threshold-based custom-written Matlab script. Analysis of changes in normalized ECoG power was performed using a custom-written Matlab script. Day 7 ECoG signals were divided into three frequency bands theta (Θ; 4–8 Hz) alpha (α; 8–13 Hz) and beta (β; 13–30 Hz) based on a fast Fourier transform algorithm with ten Blackman windows. Each window had an overlap of 50% and was calculated with 512 nodes (total duration of 7.04 s). The total power during stimulation was normalized to the corresponding baseline power (before light stimulation). For temporal precise detection of changes in theta power during Chrimson or Opn7b stimulation a 30 s window using a complex Morlet wavelet analysis with a bandwidth of 0.5 s and central frequency of 4 Hz was analyzed. Mean theta power for 5 s (5–10 s in the plot) activity was calculated for baseline activity during orange light stimulation (Chrimson), following a blue light-induced EA (Opn7b) and during orange light following a blue light-induced EA (Chrimson + Opn7b). Since the increase was equally detectable bilaterally we only analyzed the changes contralateral to the Chrimson stimulation site.

### Behavioral analysis

For behavioral testing 3–5 month old male NEX-cre mice were group-housed with an inverted light cycle. Behavioral testing was started 21 days after viral injection and were conducted between 10 am−6 pm. All mice were handled daily for 7 days before and during testing to reduce handling stress. One hour prior to the beginning of behavioral testing mice were brought to the experimental room for acclimatization. After each mouse, the arena and all objects were carefully cleaned with 70% ethanol to remove any olfactory cues. Following the behavioral tests all mice were stimulated to verify the described EA induction protocol (all mice injected with Opn7b developed EA upon blue light stimulation).

### Vertical pole test

For motor coordination and balance, mice were placed facing upwards on a grooved vertical pole (1.3 cm diameter, 60 cm length) that was secured to a platform. Latency to turn around and time to climb down on the vertical pole was recorded for each mouse. Time was stopped once all four paws were on the ground. If a mouse fell off the pole a time of 120 s was noted (during testing no mouse fell off the pole)^32,33^.

### Adhesive removal test

In order to further assess motor skills but also possible sensory deficits in the facial region an adhesive removal test was performed. After 2 min of habituation to the testing cage two differently colored adhesive stripes (Micafilm, Wero DIN13019-A; 0.3 × 0.4 cm) were attached to both front paws. The sequence in which the adhesive stripes were attached was alternated and randomly assigned to both experimental groups. The latency until recognition and removal for both sides was measured by two scientists during the test^56^.

### Open field test

To analyze general motor activity, exploration and anxiety mice were placed in the center of an acrylic glass arena (50 × 50 x 35 cm) and allowed to explore the novel arena freely for 10 min. Mice were recorded and automatically tracked with EthoVision XT 11.5 (Noldus Information Technology). Velocity, distance moved and time spent in the center and border were automatically analyzed. The arena was brightly illuminated (440–540 lux) and divided in border (10 cm surrounding the center including the edges) and center (30 × 30 cm)^6^.

### Three-chamber social interaction test

To test general sociability, mice underwent the three-chamber social interaction test. A brightly illuminated (300–490 lux) acrylic glass arena was divided into three equally sized chambers (left, right and center, 20 × 40 × 36 cm, total size 60 × 40 × 36 cm). To habituate the mice to the novel context, they were placed in the center and allowed to freely explore the arena for 10 min. After habituation, mice were guided in the center and two small wire cages were placed in the corner of the right and left chamber. One containing an unfamiliar mouse (young male < 5 weeks old) and the other a novel object (a movable table tennis ball). A sniffing zone (2 cm around the wire cage) was defined and used as an indicator for direct interaction. Then mice were allowed to freely explore the chambers for another 10 min (test phase). The chamber containing the mouse was alternated and randomly assigned to both experimental groups. The unfamiliar mice were habituated for one week to the wired cages, alternated between trials and randomly assigned to both experimental groups. During habituation and testing, mice were video recorded and tracked using EthoVision XT 11.5 (Noldus Information Technology). Duration of the time spent in each chamber and duration of the nose point in the predefined sniffing zone (2 cm) were automatically analyzed by EthoVision XT 11.5.

### Object displacement test

Mice were exposed to the object recognition test to assess spatial recognition and short-term memory. During training mice were allowed to explore the dimly illuminated (5–8 lux) acrylic glass arena (40 × 40 × 36 cm) for 10 min. Mice could only enter the arena from one side helping them to orientate inside the arena. Two identical objects (50 ml cell culture flask filled with sand, Cellstar®, Greiner Bio one, Germany) were placed equidistant from the wall inside the arena facing to the center. After the training phase mice were placed individually in a familiar cage for 2 min and one object was relocated inside the arena. The side for relocation was alternated and randomly assigned to both experimental groups. During the testing phase the mice were allowed to explore the arena for 5 min. Videos of the training and testing were manually analyzed for exploratory behavior independently by two scientists blinded for the experimental groups. A mouse was considered as exploring the object if the head was directed toward the object and the snout was close (<0.5 cm) to the object. Further criteria were the active interaction of the object (e.g., sniffing). Repetitive behavior such as biting was excluded as well as walking over the object without further interaction. A discrimination index ((Object_relocation_ − Object)/(Object_relocation_ + Object) * 100) was calculated for training and test phase. If the discrimination index in the training exceeded 20 a mouse was excluded from the analysis (1 mouse was excluded during analysis in the Opn7b group).

### Statistics and reproducibility

Statistical significance, test procedure and number of cells/animals and/or trials performed (n) are specified in the figure and/or figure legends. Statistical significance in all experiments was evaluated using SigmaPlot software v12.5 (Systat Software), Igor Pro software (v6.36, WaveMetrics), Matlab or Microsoft Excel (Office 365). For all results, the level of significance was set to *p* < 0.05 and corrected for multiple comparison as described in the figure legends. Histological experiments were performed on three or more different animals with similar results.

### Reporting summary

Further information on research design is available in the Nature Research Reporting Summary linked to this article.

## Supplementary information

Supplementary Information

Peer Review File

Reporting Summary

## Data Availability

The data that support the findings of this study are available from the corresponding author upon reasonable request. Source data are provided with this paper. The vector pAAV-CW3SL-eGFP (GenBank accession number: KJ411916.2) and the zebrafish Opn7b (*Danio rerio* novopsin-4, GenBank accession number: KT008418.1, as submitted to GenBank in 2015^13^) DNA are publicly available. Source data are provided with this paper.

## Source data

Source Data

## Code availability

All custom codes that were used for analysis are available upon reasonable request.
