# Supplementary material for: Reverse optogenetics of G protein signaling by zebrafish non-visual opsin Opn7b for synchronization of neuronal networks
Source: Nat Commun. 2021 Jul 23;12:4488. doi: 10.1038/s41467-021-24718-0 (PMC8302595; doi:10.1038/s41467-021-24718-0)
Supplement: Supplementary file 1 — Supplementary Information [file 41467_2021_24718_MOESM1_ESM.pdf]

Supplementary Information

Reverse optogenetics of G protein signaling by zebrafish non-visual opsin Opn7b for synchronization of neuronal networks

Raziye Karapinar*[1,2,3], Jan Claudius Schwitalla*[4], Dennis Eickelbeck*[1,2,3], Johanna Pakusch[4], Brix Mücher[1], Michelle Grömmke[4], Tatjana Surdin[1], Thomas Knöpfel[2], Melanie D. Mark[4#], Ida Siveke[1,5], Stefan Herlitze[1#]

[1]Department of Zoology and Neurobiology, Ruhr-University Bochum, Bochum, Germany
[2]Laboratory of Optogenetics and Circuit Neuroscience, Imperial College London, United-Kingdom
[3]The Francis Crick Institute, 1 Midland Road, London NW1 1AT, United-Kingdom
[4]Behavioral Neuroscience, Ruhr-University Bochum, Bochum, Germany
[5]German Cancer Consortium (DKTK/DKFZ), West German Cancer Center, University Hospital Essen, Hufelandstr. 55, 45147 Essen, Germany

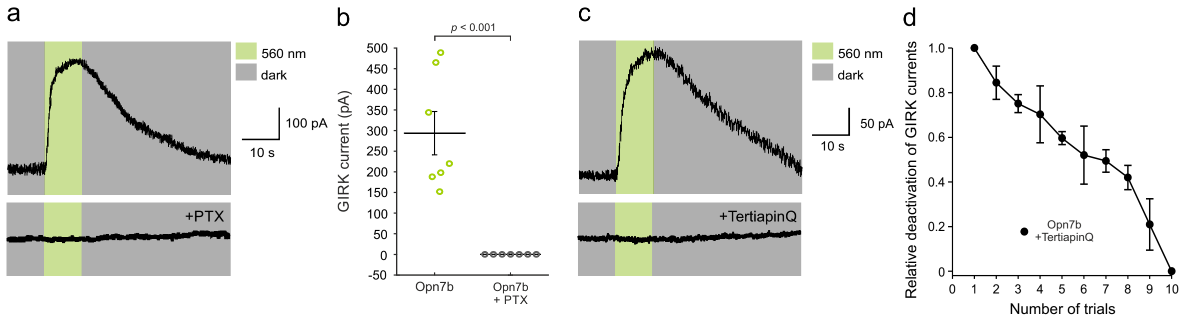

a
560 nm
dark
100 pA
10 s
+PTX
b
p < 0.001
GIRK current (pA)
Opn7b
Opn7b + PTX
c
560 nm
dark
50 pA
10 s
+TertiapinQ
d
Relative deactivation of GIRK currents
Opn7b +TertiapinQ
Number of trials

Supplementary Figure 1: *In vitro* characterization of Opn7b G protein signaling pathway specificity (related to Figure 1).
(a) Patch-clamp recording example traces of GIRK currents of one cell for Opn7b with or without bath application of pertussis toxin (PTX). **(b)** Comparison of GIRK current amplitude for Opn7b with (n = 7 cells) or without (n = 7 cells) bath application of pertussis toxin (PTX). Mean values (± SEM) and single-cell data (circles) are shown (two-sided Mann-Whitney-U-test). **(c)** Patch-clamp recording example traces of GIRK currents for Opn7b with or without bath application of TertiapinQ. **(d)** Comparison of relative GIRK current amplitude for Opn7b with or without bath application of TertiapinQ. Mean values (± SEM) are shown (n = 3 cells). Source data are provided as a Source Data file.

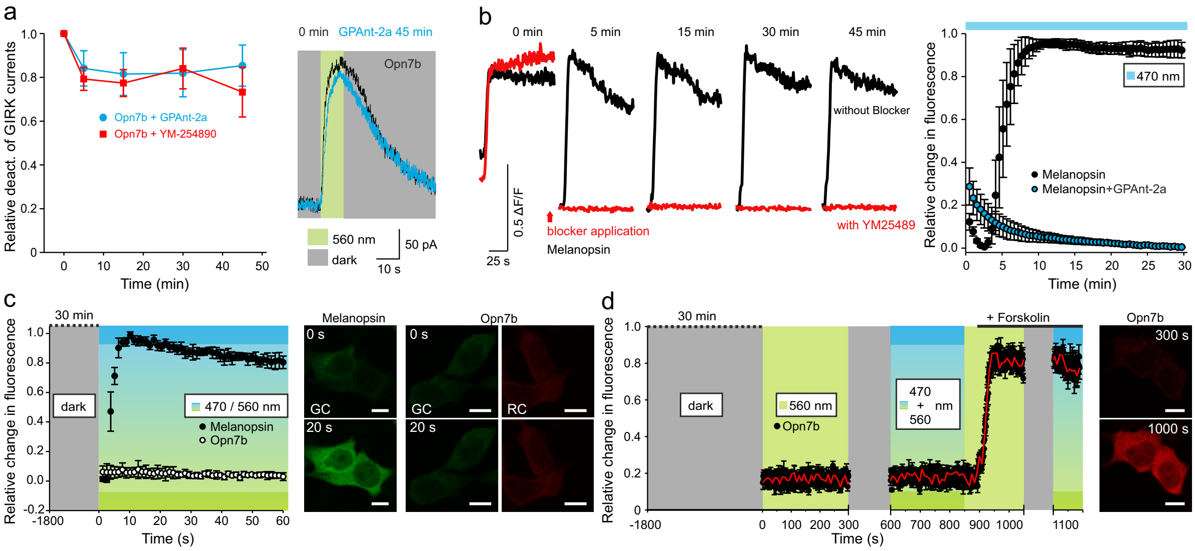
a
Relative deact. of GIRK currents
Opn7b + GPAnt-2a
Opn7b + YM-254890
Time (min)
0 min
GPAnt-2a 45 min
Opn7b
560 nm
dark
50 pA
10 s
b
0 min
5 min
15 min
30 min
45 min
without Blocker
0.5 ΔF/F
25 s
blocker application
Melanopsin
with YM25489
470 nm
Melanopsin
Melanopsin+GPAnt-2a
Relative change in fluorescence
Time (min)
c
30 min
dark
470 / 560 nm
Melanopsin
Opn7b
Relative change in fluorescence
Time (s)
Melanopsin
Opn7b
0 s
20 s
GC
RC
d
30 min
dark
560 nm
Opn7b
470
+ nm
560
+ Forskolin
Relative change in fluorescence
Time (s)
Opn7b
300 s
1000 s

Supplementary Figure 2: *In vitro* characterization of Opn7b G protein signaling pathway specificity (related to Figure 1).
(a) Comparison of relative GIRK current amplitude for Opn7b under pipette-mediated (intracellular) application of GPAnt-2a or bath application of YM-254890 at different time points after establishment of whole-cell configuration. Mean values (± SEM) are shown. (Opn7b GPAnt-2a n = 5 cells, Opn7b YM-254890 n = 7 cells, left). Patch-clamp recording example traces of GIRK currents for Opn7b under pipette-mediated (intracellular) application of GPAnt-2a at different time points after establishment of whole-cell configuration (right). **(b)** Example time course of Ca^{2+} responses for melanopsin in HEK tsA201 cells under light stimulation at different time points before and after bath application of YM-254890. Ca^{2+} signals were measured via co-expression of the genetically encoded green Ca^{2+} indicator GCaMP6m (GC, left). Time course of Ca^{2+} responses for melanopsin in HEK tsA201 cells under light stimulation with the indicated wavelength before and after pipette-mediated (intracellular) application of GPAnt-2a. Ca^{2+} signals were measured via co-expression of GC. Mean values (± SEM) are shown (n = 3 cells per group, right). **(c)** Time course of G_q-mediated Ca^{2+} responses for Opn7b and melanopsin in HEK tsA201 cells in darkness and under light stimulation with the indicated wavelengths. Ca^{2+} signals were measured via co-expression of the green/red genetically encoded Ca^{2+} indicators GC or jRCaMP1b (RC). Mean values (± SEM) are shown (n = 5 cells per group). Scale bar 10 µm. **(d)** Time course of G_s-mediated intracellular cAMP increase for Opn7b in HEK tsA201 cells in darkness and under light stimulation with the indicated wavelengths. Increase of intracellular cAMP was measured via co-expression of the red cAMP indicator Pink Flamindo. Expressing cells were stimulated with forskolin in a final step. Scale bar 10 µm. Mean values (± SEM) are shown (n = 5 cells per group). Source data are provided as a Source Data file.

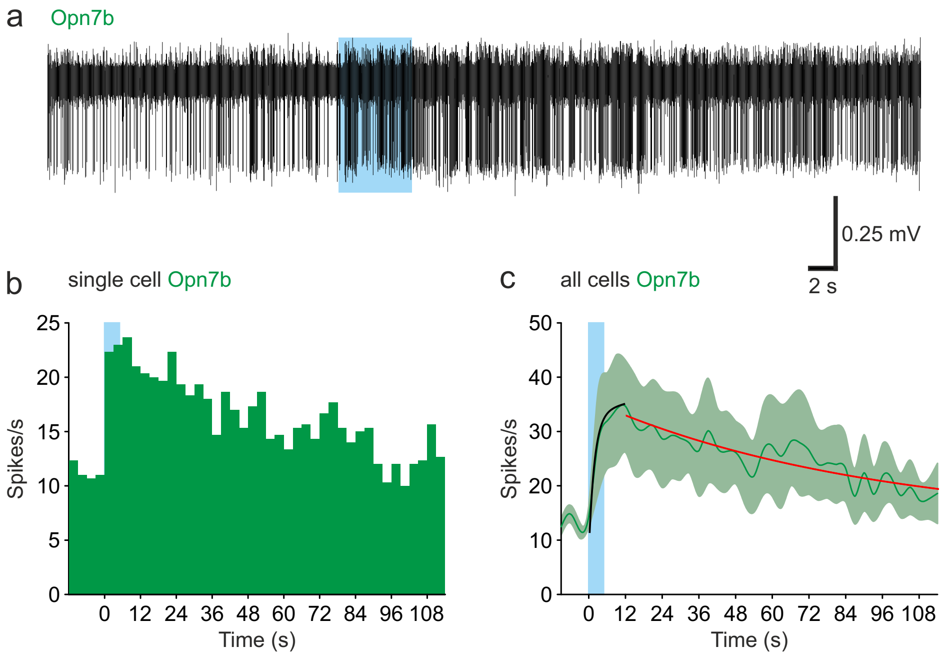
a
Opn7b
0.25 mV
2 s
b
single cell Opn7b
Spikes/s
Time (s)
c
all cells Opn7b
Spikes/s
Time (s)

Supplementary Figure 3: Opn7b activation can be long-lasting (related to Figure 3).
(a) Representative example of long-lasting single-cell activity change after blue light (465 nm, blue box) stimulation in Opn7b (green) expressing cells in the S1 of a Bl6 mouse. **(b)** Cells with a higher baseline firing activity show a long-lasting activity increase after 5 s stimulation. Representative example of a single cell. Bin size 3 s. **(c)** Mean activity change after 5 s blue light stimulation (n = 5 cells from 1 animal). Time constants of activation (τ_{light} = 2.4 s, black line) and recovery ($\tau_{recovery}$ = 90.87 s, red line) of light-induced maximum firing rate are shown. Green line represents mean while shadowed area represents the ± SEM. Source data are provided as a Source Data file.

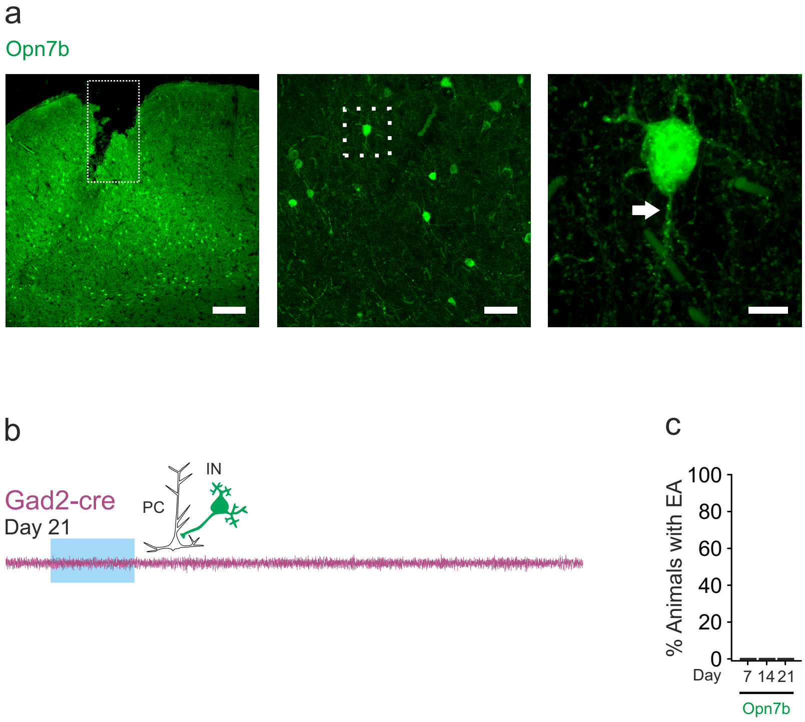
a
Opn7b
b
Gad2-cre
Day 21
PC
IN
c
% Animals with EA
100
80
60
40
20
0
Day
7
14
21
Opn7b

Supplementary Figure 4: Light stimulation of Opn7b in GABAergic interneurons of the primary somatosensory cortex does not induce epileptiform activity (related to Figure 4).

(a) Representative example of floxed Opn7b (green) expression in the primary somatosensory cortex of Gad2-cre mice. Dashed box represents the fiber tract of the implanted fiber (left). The dashed box in the middle panel represents the zoom of a single neuron and its axon (arrow) in the right panel. Scale bar 200 µm, 50 µm, 10 µm. **(b)** Opn7b was exclusively expressed in GABAergic interneurons by using Gad2-cre animals. Example trace of electrocorticogram activity after 10 s blue light (465 nm, blue box) stimulation on day 21 post injection induced no epileptiform activity (EA). **(c)** None of the tested Gad2-cre (n = 3) animals developed EA following 10 s blue light stimulation on day 7-21. Pyramidal cell (PC); Interneuron (IN). Source data are provided as a Source Data file.

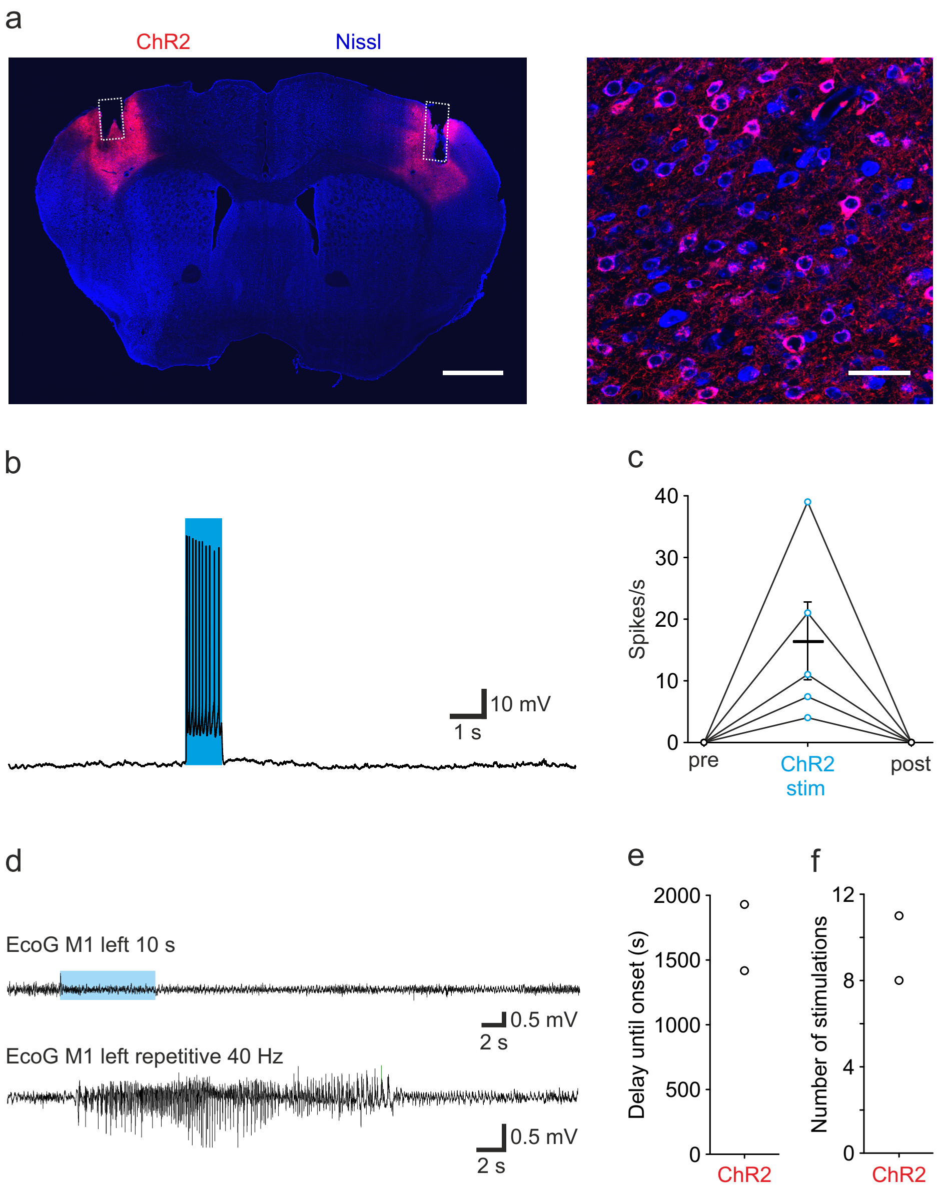

a
ChR2
Nissl
b
10 mV
1 s
c
40
30
20
10
0
Spikes/s
pre
ChR2
stim
post
d
EcoG M1 left 10 s
0.5 mV
2 s
EcoG M1 left repetitive 40 Hz
0.5 mV
2 s
e
2000
1500
1000
500
0
Delay until onset (s)
ChR2
f
12
8
4
0
Number of stimulations
ChR2

Supplementary Figure 5: Channelrhodopsin-2 activation induces robust action potential firing and seizure onset after repetitive long-term light stimulation (related to Figure 4).

(a) Coronal section and zoom of NEX-cre mouse bilaterally expressing floxed channelrhodopsin-2 (ChR2, red) in the primary somatosensory cortex (S1) containing a neuronal staining (Nissl, blue). The dashed boxes represent fiber tracts of the implanted fibers. Scale bar 1 mm and 50 µm. **(b)** and **(c)** Representative current-clamp recording in S1 of NEX-cre mouse expressing ChR2. Blue light (1 s, 465 nm, blue box) stimulation induced action potential firing (n = 5 cells from 1 animal). Each circle represents individual cells, while the bar represents the mean value for all cells. Error bars represented as ± SEM. **(d)** Representative example of 10 s blue light (465 nm) stimulation and repetitive 40 Hz ChR2 blue light stimulation that triggered epileptiform activity (EA). **(e)** Delay and **(f)** number of stimulations based on the first stimulation until onset of EA after repetitive 40 Hz stimulation (n = 2, 3 tested animals). Each circle represents EA of individual animals. Source data are provided as a Source Data file.

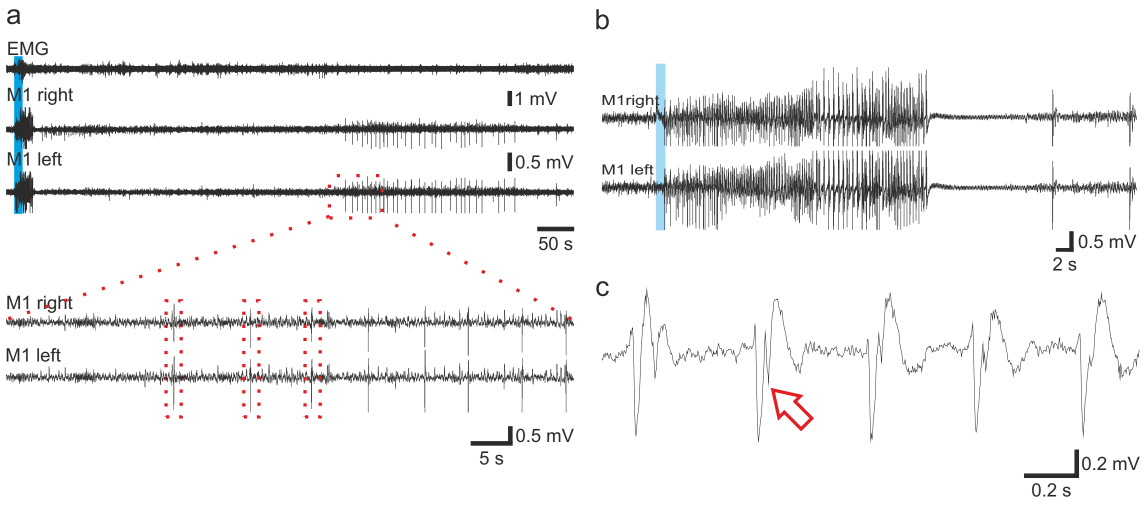

a
EMG
M1 right
1 mV
M1 left
0.5 mV
50 s
M1 right
M1 left
0.5 mV
5 s
b
M1right
M1 left
0.5 mV
2 s
c
0.2 mV
0.2 s

Supplementary Figure 6: Characteristic features of ictal and postictal electrocorticographic changes in Opn7b expressing animals related to Figure 4).

(a) Interictal events characterized by high amplitude spikes were detectable in Opn7b expressing animals only shortly after stimulation (blue box) and induction of generalized epileptiform activity (EA). Depicted is a NEX-cre mouse day 14 after 10 s stimulation. Bottom panel shows individual interictal events (dashed box). Primary motor cortex (M1), electromyogram (EMG) **(b)** A part of the EA showed a distinct postictal suppression. Depicted is a NEX-cre mouse after 1 s stimulation. **(c)** Polyspikes (red arrow) were sometimes detectable in the ECoG reording during EA.

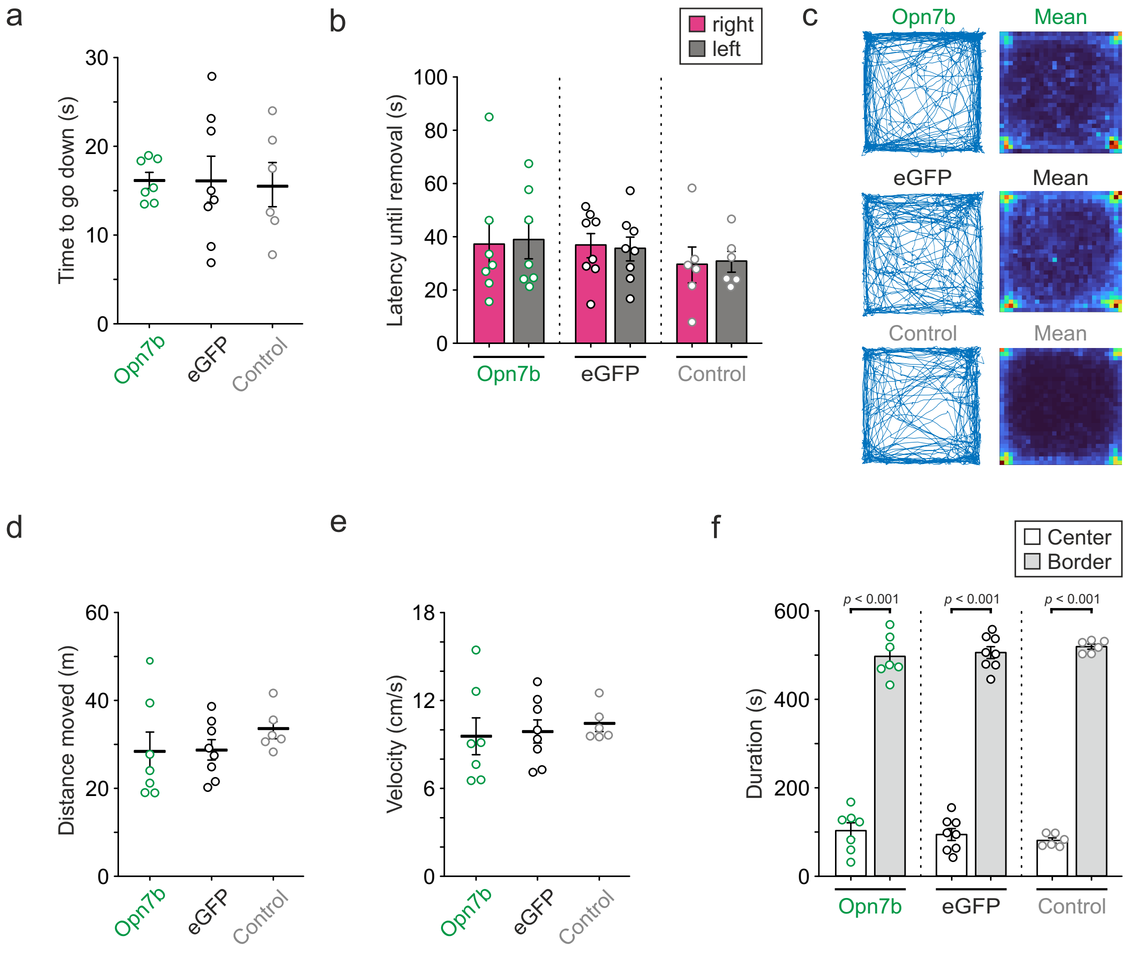

a
b
c
d
e
f
Time to go down (s)
Latency until removal (s)
right
left
Opn7b
eGFP
Control
Mean
Distance moved (m)
Velocity (cm/s)
Duration (s)
Center
Border
p < 0.001

Supplementary Figure 7: Mice expressing Opn7b in pyramidal cells of the primary somatosensory cortex show no changes in motor and sensory skills (related to Figure 4).

(a) No significant changes were found in the pole test between NEX-cre mice injected with floxed Opn7b (green, n = 7) or floxed enhanced green fluorescent protein (eGFP, black, n = 8) and control animals (grey, not injected, n = 6) for time to go down on the pole as well as **(b)** latency until the removal of two adhesive stripes from the front right and left paws in the adhesive removal test. Floxed Opn7b n = 7, floxed eGFP n = 8 and control n = 6. **(c)** Representative individual examples as well as mean duration in the different areas of the arena during the 15 min for all animals per group in the open field test. **(d)** No significant difference was found for distance moved, **(e)** velocity and **(f)** the preference to move in the border area (grey) of the arena for NEX-cre mice injected with floxed Opn7b or floxed eGFP and control animals (floxed Opn7b $t(12) = -15.593$, floxed eGFP $t(14) = -21.615$, control $t(10) = -53.828$, two-sided independent samples *t*-test). For D-F the following numbers of animals were used floxed Opn7b (n = 7), floxed eGFP (n = 8) and control (n = 6). For A, B and D-F each circle represents individual animals, while the bar represents the mean value for all animals. Error bars represented as ± SEM. Source data are provided as a Source Data file.

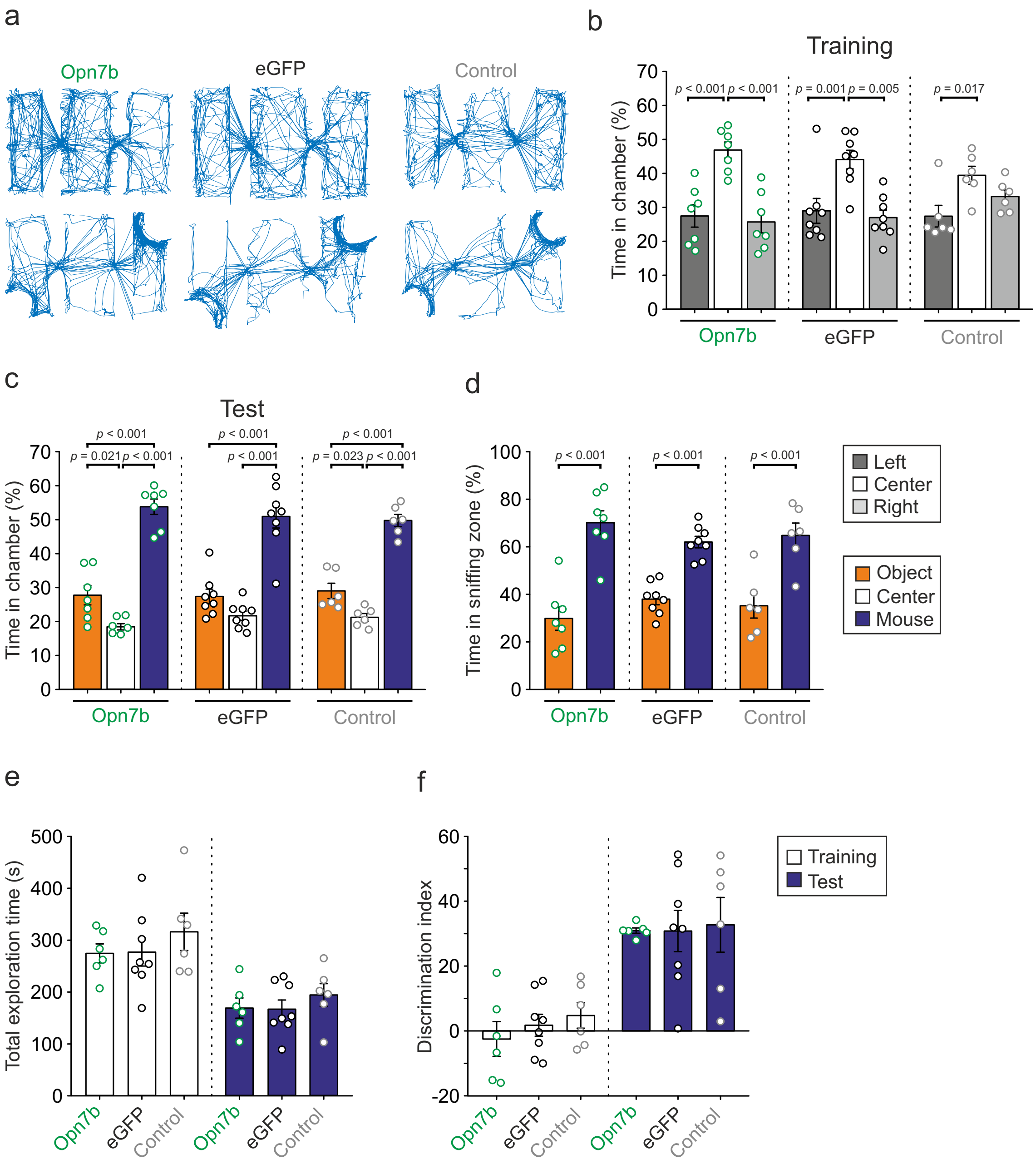
a
Opn7b
eGFP
Control
b
Training
Time in chamber (%)
p < 0.001
p < 0.001
p = 0.001
p = 0.005
p = 0.017
Opn7b
eGFP
Control
c
Test
Time in chamber (%)
p < 0.001
p = 0.021
p < 0.001
p < 0.001
p < 0.001
p < 0.001
p = 0.023
p < 0.001
Opn7b
eGFP
Control
d
Time in sniffing zone (%)
p < 0.001
p < 0.001
p < 0.001
Opn7b
eGFP
Control
Left
Center
Right
Object
Center
Mouse
e
Total exploration time (s)
Opn7b
eGFP
Control
Opn7b
eGFP
Control
f
Discrimination index
Opn7b
eGFP
Control
Opn7b
eGFP
Control
Training
Test

Supplementary Figure 8: Mice expressing Opn7b in pyramidal cells of the primary somatosensory cortex show no difference in social and cognitive skills (related to Figure 4).

(a) Representative examples of the three-chamber social interaction test during training and test phase for NEX-cre mice injected with floxed Opn7b (green) or floxed enhanced green fluorescent protein (eGFP, black) and control (grey, uninjected) animals. **(b)** All groups (floxed Opn7b n = 7, floxed eGFP n = 8 and control n = 6) tested spent significantly more time in the center during training (floxed Opn7b $p < 0.001$ $F(2,18) = 15.556$, floxed eGFP $p < 0.001$ $F(2,21) = 10.128$, control $p = 0.019$ $F(2,15) = 5.196$, one-way ANOVA followed by pairwise Bonferroni post hoc test) and **(c)** significantly more time in the chamber with the mouse during the test phase in the social interaction test (floxed Opn7b p < 0.001 $F(2,18) = 71.601$, floxed eGFP p < 0.001 $F(2,21) = 39.959$, control p < 0.001 $F(2,15) = 68.161$, one-way ANOVA followed by pairwise Bonferroni post hoc test, floxed Opn7b n = 7, floxed eGFP n = 8 and control n = 6). **(d)** All groups (floxed Opn7b n = 7, floxed eGFP n = 8 and control n = 6) explored the 2 cm sniffing zone around the social interaction partner significantly longer than the novel object (floxed Opn7b $t(12) = -40.276$, floxed eGFP $t(14) = -23.986$, control $t(10) = -29.602$, two-sided independent samples *t*-test). **(e)** No difference was found in the object displacement test for the total exploration time during training or test phase in animals injected with floxed Opn7b (n = 6) or floxed eGFP (n = 8) and control (n = 6) animals. **(f)** All groups showed a discrimination index around 30 during testing, which indicates intact short-term memory skills. Floxed Opn7b n = 6, floxed eGFP n = 8 and control n = 6. For B-D, F and G each circle represents individual animals, while the bar represents the mean value for all animals. Error bars represented as ± SEM. Source data are provided as a Source Data file.

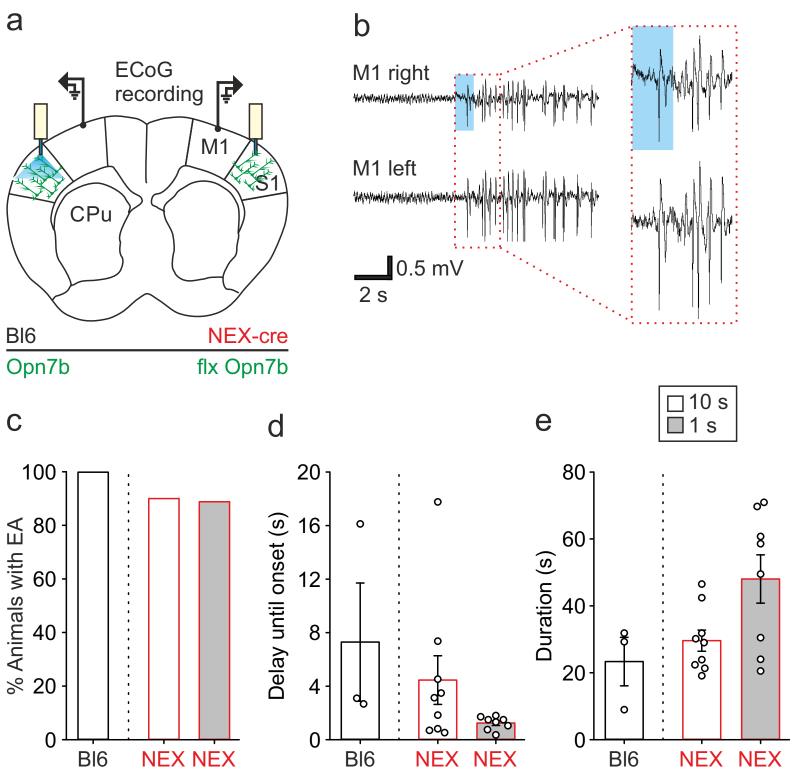
a
ECoG
recording
M1
S1
CPu
Bl6
NEX-cre
Opn7b
flx Opn7b
b
M1 right
M1 left
0.5 mV
2 s
c
% Animals with EA
100
80
60
40
20
0
Bl6
NEX NEX
d
Delay until onset (s)
20
16
12
8
4
0
Bl6
NEX
NEX
e
10 s
1 s
Duration (s)
80
60
40
20
0
Bl6
NEX
NEX

Supplementary Figure 9: Unilateral stimulation of Opn7b induces generalized epileptiform activity (related to Figure 5).

(a) Unilateral blue light (465 nm) stimulation of bilateral injected animals. NEX-cre or Bl6 mice were connected to a single optic fiber during electrocorticographic (ECoG) recordings. **(b)** Example trace of unilateral stimulation shows a clear bilateral beginning of the epileptiform activity (EA) after unilateral stimulation. **(c)** All of the Bl6 mice (n = 3) developed EA after 10 s unilateral blue light stimulation. 90% of NEX-cre developed EA after 10 s (n = 10) and 88% after 1 s (n = 9) unilateral blue light stimulation. **(d)** Delay until onset and **(e)** duration of EA after unilateral stimulation in Bl6 and NEX-cre mice (Bl6 n = 3, NEX-cre 10 s n = 9, NEX-cre 1 s n = 8). For D and E each circle represents individual animals, while the bar represents the mean value for all animals. Error bars represented as ± SEM. Source data are provided as a Source Data file.

Supplementary Table 1

Primer Name	Nucleotide Sequence
Forward Opn7b	5'-GATAAGCTTGATTCGAGCTAGCACAAGTTTGTACAAAA-3'
Reverse Opn7b	5'-CGACCGGTGGATCCCGGGCCCGCGGCAGAAAG-3'
Forward Nex-Cre	5'-GAGTCCTGGAATCAGTCTTTTTC-3'
Reverse Nex-Cre	5'-CCGCATAACCAGTGAAACAG-3'
Forward Nex	5'-GAGTCCTGGAATCAGTCTTTTTC-3'
Reverse Nex	5'-AGAATGTGGAGTAGGGTGAC-3'
Forward Gad2-Cre	5'-CTTCTTCCGCATGGTCATCT-3'
Reverse Gad2-Cre	5'-CACCCCACTGGTTTTGATTT-3'
Forward Gad2	5'-CACCCCACTGGTTTTGATTT-3'
Reverse Gad2	5'-AAAGCAATAGCATCACAAATTTCA-3'

Primers used to PCR amplify and clone Opn7b and to determine the genetic background of the mouse lines used.
