# Supplementary material for: Reverse optogenetics of G protein signaling by zebrafish non-visual opsin Opn7b for synchronization of neuronal networks
Source: Nat Commun. 2021 Jul 23;12:4488. doi: 10.1038/s41467-021-24718-0 (PMC8302595; doi:10.1038/s41467-021-24718-0)
Supplement: Supplementary file 3 — Reporting Summary [file 41467_2021_24718_MOESM3_ESM.pdf]

nature research

Corresponding author(s): Stefan Herlitze and Melanie D. Mark

Last updated by author(s): Jun 14, 2021

Reporting Summary

Nature Research wishes to improve the reproducibility of the work that we publish. This form provides structure for consistency and transparency in reporting. For further information on Nature Research policies, see our Editorial Policies and the Editorial Policy Checklist.

Statistics

For all statistical analyses, confirm that the following items are present in the figure legend, table legend, main text, or Methods section.

n/a	Confirmed	
☐	☒	The exact sample size (n) for each experimental group/condition, given as a discrete number and unit of measurement
☐	☒	A statement on whether measurements were taken from distinct samples or whether the same sample was measured repeatedly
☐	☒	The statistical test(s) used AND whether they are one- or two-sided<br>*Only common tests should be described solely by name; describe more complex techniques in the Methods section.*
☒	☐	A description of all covariates tested
☐	☒	A description of any assumptions or corrections, such as tests of normality and adjustment for multiple comparisons
☐	☒	A full description of the statistical parameters including central tendency (e.g. means) or other basic estimates (e.g. regression coefficient) AND variation (e.g. standard deviation) or associated estimates of uncertainty (e.g. confidence intervals)
☐	☒	For null hypothesis testing, the test statistic (e.g. F, t, r) with confidence intervals, effect sizes, degrees of freedom and P value noted<br>*Give P values as exact values whenever suitable.*
☒	☐	For Bayesian analysis, information on the choice of priors and Markov chain Monte Carlo settings
☒	☐	For hierarchical and complex designs, identification of the appropriate level for tests and full reporting of outcomes
☒	☐	Estimates of effect sizes (e.g. Cohen's d, Pearson's r), indicating how they were calculated

Our web collection on statistics for biologists contains articles on many of the points above.

Software and code

Policy information about availability of computer code

Data collection	Spike 2 (v7), Leica Application Suite (for Leica TCS SP5 confocal laser scanning microscope); HEKA PatchMaster software (v2x52), Inscopix recording software, Ethovision XT 11.5 (Noldus Information Technology)
Data analysis	Matlab 2019b, Igor Pro software (v6.36,WaveMetrics); Microsoft Excel (Office 365); SigmaPlot software v12.5 (Systat Software), Inscopix Data processing software, Ethovision XT 11.5 (Noldus Information Technology), Image J (v1.52o)

For manuscripts utilizing custom algorithms or software that are central to the research but not yet described in published literature, software must be made available to editors and reviewers. We strongly encourage code deposition in a community repository (e.g. GitHub). See the Nature Research guidelines for submitting code & software for further information.

Data

Policy information about availability of data

All manuscripts must include a data availability statement. This statement should provide the following information, where applicable:
- Accession codes, unique identifiers, or web links for publicly available datasets
- A list of figures that have associated raw data
- A description of any restrictions on data availability

The data that support the findings of this study are available from the corresponding author upon reasonable request. Source data are provided with this paper.The vector pAAV-CW3SL-eGFP (GenBank accession number: KJ411916.2) and the zebrafish Opn7b (Danio rerio novopsin-4, GenBank accession number: KT008418.1, as submitted to GenBank in 201513) DNA are publicly available.

April 2020

Field-specific reporting

Please select the one below that is the best fit for your research. If you are not sure, read the appropriate sections before making your selection.

- [x] Life sciences
- [ ] Behavioural & social sciences
- [ ] Ecological, evolutionary & environmental sciences

For a reference copy of the document with all sections, see nature.com/documents/nr-reporting-summary-flat.pdf

Life sciences study design

All studies must disclose on these points even when the disclosure is negative.

Sample size	No statistical methods were used to determine sample size before experiments. Sample size were based on the follwing publications Khoshkhoo et al. 2017 (10.1016/j.neuron.2016.11.043), Berry et al. 2019(10.1038/s41467-019-09124-x), Chen et al. 2021 (10.1038/s41587-020-0679-9)
Data exclusions	3 mice with misplaced viral-injection or optic fiber implantation verified by histological analysis were excluded. In the object displacement test 1 mouse was excluded during analysis in the Opn7b group because the discrimination index (Objectrelocation-Object)/(Objectrelocation +Object)*100) in the training exceeded 20. Otherwise, no data were excluded.
Replication	Results were replicated in different mice and cells, the exact number of tested animals and cells is stated in the corresponding figure legends.
Randomization	In Hek cells the wavelength and duration of the light pulse that was given was randomized.<br>For all tests involving mice the animals were randomly allocated in each experimental group.<br>For the adhesive removal test the sequence in which the adhesive stripes were attached was alternated and randomly assigned to both experimental groups.<br>For the three chamber social interaction test the chamber containing the mouse was alternated and randomly assigned to both experimental groups. The unfamiliar mice were habituated for one week to the wired cages, alternated between trials and randomly assigned to both experimental groups.<br>For the object displacement test the side for relocation was alternated and randomly assigned to both experimental groups.
Blinding	Investigators were blinded for the viral construct (Opn7b, ChR2 or eGFP) expressed in the mice during the analysis of electrocorticogram recordings and behavior experiments. During calcium imaging and chrimson stimulation investigators were not blinded since all animals expressed the same viral construct. For experiments in hek cells investigators were not blinded.

Reporting for specific materials, systems and methods

We require information from authors about some types of materials, experimental systems and methods used in many studies. Here, indicate whether each material, system or method listed is relevant to your study. If you are not sure if a list item applies to your research, read the appropriate section before selecting a response.

Materials & experimental systems

n/a	Involved in the study	
x		Antibodies
	x	Eukaryotic cell lines
x		Palaeontology and archaeology
	x	Animals and other organisms
x		Human research participants
x		Clinical data
x		Dual use research of concern

Methods

n/a	Involved in the study	
x		ChIP-seq
x		Flow cytometry
x		MRI-based neuroimaging

Eukaryotic cell lines

Policy information about cell lines

Cell line source(s)	Human embryonic kidney (HEK) tsA201 cells (Sigma Aldrich, Taufkirchen, Germany) and HEK GIRK 1/2 cells (HEK293 cells stably expressing GIRK1/2 subunits, kindly provided by Dr. A. Tinker UCL London, GB).
Authentication	Cell line (HEK tsA201) authentication was conducted by supplier (Sigma Aldrich, Taufkirchen, Germany). GIRK expressing cell line was characterized using electrophysiological methods (IV curve)
Mycoplasma contamination	All cell lines tested negative for mycoplasma contamination.
Commonly misidentified lines (See ICLAC register)	No commonly misidentified cell lines were used.

Animals and other organisms

Policy information about studies involving animals; ARRIVE guidelines recommended for reporting animal research

Laboratory animals	2-6 month old male and female C57Bl6/J (JAX stock no. 000664); heterozygous Nex-Cre (DOI: 10.1002/dvg.20256); heterozygous Gad65-Ires-Cre mice (Gad2tm2(cre)Zjh/J), JAX stock no. 010802
Wild animals	The study did not involve wild animals
Field-collected samples	The study did not involve field-collected samples
Ethics oversight	All experiments were conducted with approval of a local ethics committee (Bezirksamt Arnsberg) and the animal care committee of Nordrhein-Westfalen (LANUV; Landesamt für Umweltschutz, Naturschutz und Verbraucherschutz Nordrhein-Westfalen, Germany; AZ. 84-02.04.2014.A203 und AZ. 81-02.04.2019.A228). The study was carried out in accordance with the European Communities Council Directive of 2010 (2010/63/ EU) for care of laboratory animals and supervised by the animal welfare commission of the Ruhr-University Bochum.

Note that full information on the approval of the study protocol must also be provided in the manuscript.
