# Supplementary material for: Reverse optogenetics of G protein signaling by zebrafish non-visual opsin Opn7b for synchronization of neuronal networks
Source: Nat Commun. 2021 Jul 23;12:4488. doi: 10.1038/s41467-021-24718-0 (PMC8302595; doi:10.1038/s41467-021-24718-0)
Supplement: Supplementary file 2 — Peer Review File [file 41467_2021_24718_MOESM2_ESM.pdf]

Editorial Note: This manuscript has been previously reviewed at another journal that is not operating a transparent peer review scheme. The manuscript was considered suitable for publication without further review at *Nature Communications.*
